# Emerging concepts in designing next-generation multifunctional nanomedicine for cancer treatment

**DOI:** 10.1042/BSR20212051

**Published:** 2022-07-08

**Authors:** Kasturee Chakraborty, Archana Tripathi, Sukumar Mishra, Argha Mario Mallick, Rituparna Sinha Roy

**Affiliations:** 1 Department of Biological Sciences, Indian Institute of Science Education and Research Kolkata, Mohanpur 741246, India; 2 Centre for Advanced Functional Materials, Indian Institute of Science Education and Research Kolkata, Mohanpur 741246, India; 3 Centre for Climate and Environmental Studies, Indian Institute of Science Education and Research Kolkata, Mohanpur 741246, India

**Keywords:** Cancer, Combination therapy, Immuno therapy, Nanomedicine, peptide

## Abstract

Nanotherapy has emerged as an improved anticancer therapeutic strategy to circumvent the harmful side effects of chemotherapy. It has been proven to be beneficial to offer multiple advantages, including their capacity to carry different therapeutic agents, longer circulation time and increased therapeutic index with reduced toxicity. Over time, nanotherapy evolved in terms of their designing strategies like geometry, size, composition or chemistry to circumvent the biological barriers. Multifunctional nanoscale materials are widely used as molecular transporter for delivering therapeutics and imaging agents. Nanomedicine involving multi-component chemotherapeutic drug-based combination therapy has been found to be an improved promising approach to increase the efficacy of cancer treatment. Next-generation nanomedicine has also utilized and combined immunotherapy to increase its therapeutic efficacy. It helps in targeting tumor immune response sparing the healthy systemic immune function. In this review, we have summarized the progress of nanotechnology in terms of nanoparticle designing and targeting cancer. We have also discussed its further applications in combination therapy and cancer immunotherapy. Integrating patient-specific proteomics and biomarker based information and harnessing clinically safe nanotechnology, the development of precision nanomedicine could revolutionize the effective cancer therapy.

## Introduction

Cancer remains the most fatal and life-threatening disease worldwide, causing an estimated 19.3 million new cases and 10.0 million deaths in 2020 [1]. Current therapeutic approaches are not found to completely cure the advanced cancer having distant organ metastases. Treatment of cancer follows several approaches, including locoregional (surgery and radiation therapy), chemotherapy, endocrine (hormone) therapy, targeted therapy, etc. Such therapeutic approaches also possess certain disadvantages such as fatigue, numbness, nail changes, hair loss, loss of appetite, mouth sores, nausea, weight changes, vomiting, diarrhoea, heart damage, etc [2–6]. In spite of its huge potential, chemotherapy remains disadvantageous in having off-target side effects and non-specific delivery [7]. Nanotechnology has become a promising approach to overcome this limitation. The nano-sized materials provide opportunities for their use in diagnosing, monitoring, controlling, preventing and treating diseases [8]. The concept of nanoparticles was first adapted by Nobel laureate Richard P. Feynman in his famous lecture entitled “There’s plenty of room at the bottom” in December 1959 [9]. Nanotherapy is the therapeutic strategy harboring nanoparticles ranging from 10 to 100 nm for intravenous delivery [10,11]. Nanoparticles below 10 nm diameter are prone to renal clearance [11]. Widely used examples of nanoparticles having anticancer efficacy are Doxil, a liposomal formulation of Doxorubicin and Abraxane, an albumin-bound nanoparticle of paclitaxel [12]. These drugs also received approval from U.S. Food and Drug Administration (FDA). Apart from that, several other nanotechnology platforms like organic, inorganic and organometallic nanoparticles have also been used over the past two decades for therapeutic, diagnostic and theranostic purposes [13]. Nanoparticles can be easily custom-tailored and advantageous in having the following features: (1) carry a high payload of biologically active drug [11,14,15], (2) protects the drug from degradation, (3) contain large surface area to accommodate multiple targeting ligands (4) larger surface to interact with multiple types of drug molecules, (5) controlled release profile of drug and (6) potential to bypass multidrug resistant mechanism [11,16,17].

Nanotechnology also undergoes several barriers that hinder its successful translation into the clinic. Various biological barriers limit the functionality of the nanoparticles and their clinical outcome. Nanoparticles get easily phagocytosed and degraded by the macrophages of the liver, spleen, lungs, lymph nodes and skin [13]. According to recent statistics, 0.7% of the total injected dose can get targeted to tumor. The primary reason for this failure is the formation of protein corona (proteins adsorbed on the nanoparticle from plasma and/or intracellular fluid) around nanoparticles and their subsequent opsonization (the process where opsonins or extracellular proteins bind to the surface of the nanoparticles, causing the degradation of nanoparticles by phagocytes) and eventual phagocytosis [10,13]. High encapsulation efficiency of a drug does not always lead to high therapeutic potential. Again, hydrophobic drugs which precipitate over time also do not show significant efficacy [10]. Therefore, designing a potent yet stable nanoparticle has always been challenging. In the following section, we will focus on the strategies acquired by the researchers in designing effective nanoparticles to aid anticancer therapy and we will also describe the progress made so far with nanotherapy.

**Figure 1:**
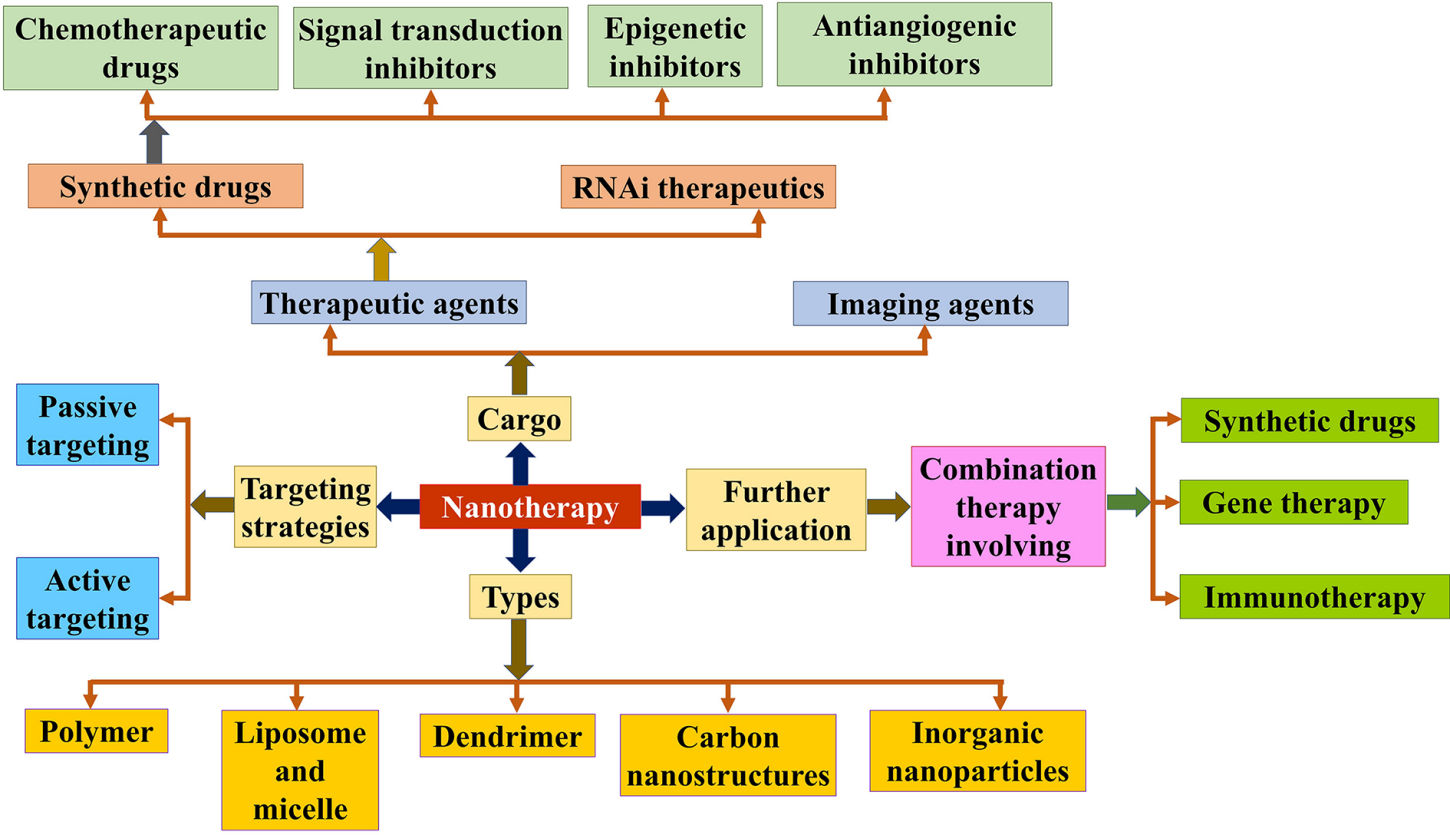
Overview of the basis of multifunctional cancer nanotherapy and its potential applications The figure describes the targeting strategies involved in nanotherapy, the composition of nanoparticles, types of cargo and its further applications in combination therapies.

By designing suitable combinatorial nanotherapeutics, one can achieve targeted delivery, reduce side effects of free drugs, delay in developing drug resistance and accomplish synergistic drug interactions at low doses [18]. Oncogenic signaling pathways can also be targeted using nanoparticles to selectively target the tumor cells without causing systemic toxicity. Moreover, nanomedicine mediated anti-angiogenic drug delivery makes them more reachable towards tumor vasculature. Furthermore, nanoparticles potentiate the gene therapy to introduce therapeutic nucleic acids into target cells to achieve curative response in cancer patients [19]. Recently, nanotherapy has also been used to deliver immunotherapeutic drugs in combination with conventional therapeutic modalities (e.g., chemotherapy, RNAi therapy, photothermal, photodynamic and radiotherapy) to increase anti-tumor efficacy [20]. Minimally invasive photothermal therapy allows killing of cancer cells by heat generated upon their exposure to the near infrared (NIR) light [20]. Similarly, photodynamic therapy utilizes photosensitizers which upon exposure to light releases reactive oxygen species and induce cellular toxicity toward tumors [20]. Radiotherapy induced radiation damage of cancer cells is widely used in cancer treatment, which allows curative treatment of 40% patients out of >50% of the patients with cancer treated by radiotherapy [20,21]. Cancer immunotherapy has gained tremendous attention for providing long-term treatment in cancer, as it facilitates immunological memory induced delay in cancer remission. One of the key challenges of implementing immunotherapy is off-target responses. Nanotherapy has potential to overcome this challenge by delivering these immunotherapeutic drugs to desired target sites [22]. In this review, we have discussed the potential applications and the strength of nanotherapy by combining therapies like chemotherapy, gene therapy and immunotherapy. The central theme of this review is on engineering multifunctional cancer nanotherapy as described in Figure 1.

## Nanoparticle designing

Engineering multifunctional nanoparticle or precision nanomedicine still remains challenging. The primary aim of nanoparticle design is to transport therapeutic drugs and imaging agents. Such cargos are loaded on to nanoparticles either by chemical conjugation or by encapsulation [7]. Researchers found that the encapsulation technique remains more effective for clinical translation of a low potency drug than chemical conjugation. In order to produce efficacy, the drug should comprise at least 10% (wt/wt) of the entire nanoparticle composition [13]. While designing nanoparticles, certain criteria are needed to be taken into consideration, such as stability, pharmacological reasonability, pathophysiological suitability, etc [13]. Current development of cancer nanomedicine has provided answers to problems like (1) prolonged blood circulation to improve stability and bioavailability, (2) adequate tumor accumulation and (3) controlled drug release and uptake by tumor cells having a release profile for specific targeting [16,17].

## Prolonged blood circulation to improve bioavailability

Proteins present in biological fluids get adsorbed in the nanoparticles when administered. Such nanoparticle-protein complexes get easily recognized by macrophages and phagocytosed [10,23,24]. To overcome this difficulty, poly(ethylene glycol) (PEG) is often used to form a hydrophilic “stealth” [25]. PEGylation promotes solubilization of the nanoparticles, prevents opsonization and increases the half-life of the drug in the blood stream [26]. Doxil (liposomal doxorubicin) is a commonly used PEGylated nanoparticle. It shows ∼100 times longer half-life compared with free doxorubicin in circulation [27]. Current research has utilized nanotechnology to increase the solubility of potent but poorly soluble drugs such as cyclosporine, paclitaxel, amphotericin B, etc [28].

## Adequate tumor accumulation

**Figure 2:**
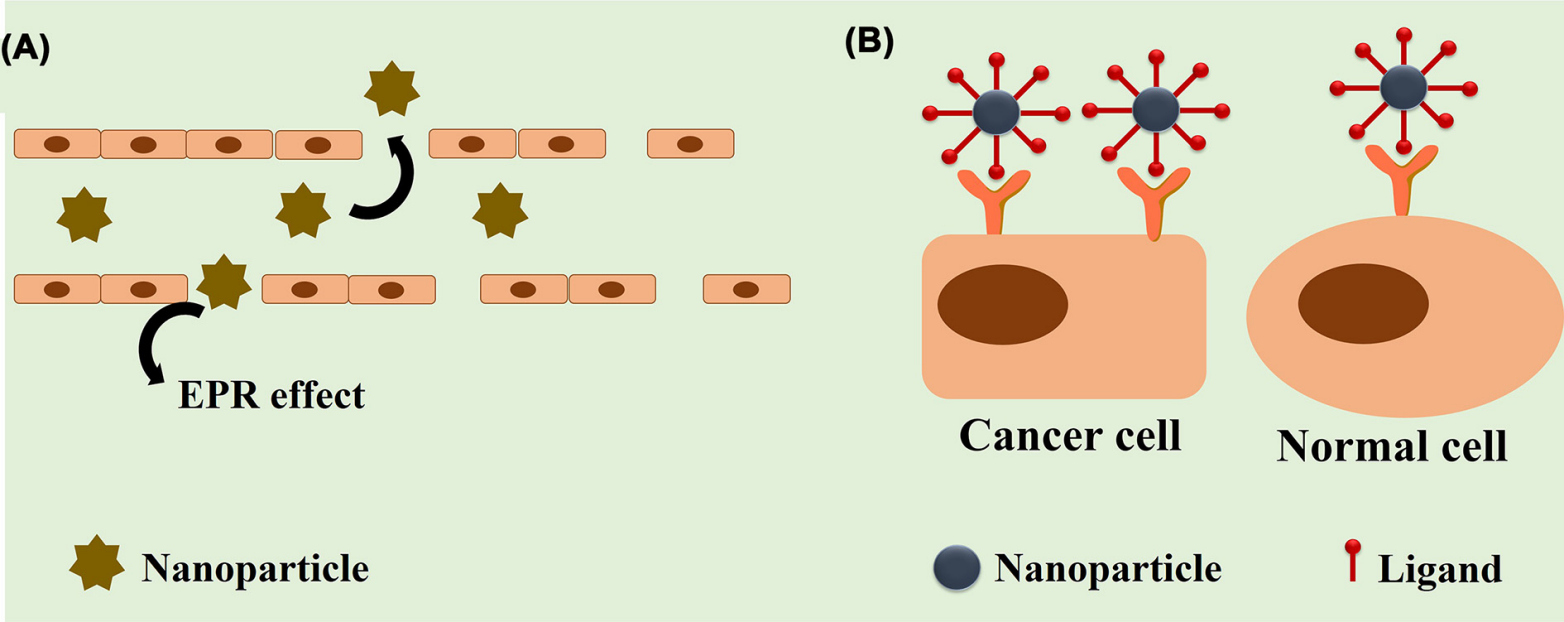
Targeting strategies of nanoparticles Figure illustrating (**A**) the passive targeting (enhanced permeability and retention effect or EPR effect) and (**B**) the active targeting into a tumor. In passive targeting, nanoparticles extravasate through the leaky blood vessels of tumor vasculature having gap sizes of 100 nm to 2 μm. Due to poor lymphatic drainage, nanoparticles home at the tumor site. In active targeting, targeting ligands are attached to the nanoparticles that specifically target cancer's overexpressed receptors. The optimal size range to perform the EPR effect is 20–200 nm.

Nanoparticles possess unique properties due to their small size termed as “enhanced permeability and retention effect” (EPR effect) (Figure 2A) [29,30]. Harboring this phenomenon, nanoparticles extravasate through the leaky blood vessels (inter endothelial gaps as large as 500 nm) of the tumor vasculature and preferably accumulate at the tumor site. Due to poor lymphatic drainage, they retain at the tumor site. This phenomenon is also termed “passive targeting” (Figure 2A). Nanotherapeutics used for passive targeting are listed in Tables 1 and 11. However, targeting nanoparticles to the tumor tissue does not always remain successful due to variable vessel permeability [7].

**Table 1:** Nanotherapeutics used for passive targeting

Name	Formulation	Diameter (nm)	Comments	Ref.
SP1049C	Pluronic micelle+DOX	22–27	Micelle nanoparticle	[11,31]
NK911	PEF-Asp micelle + DOX	40	Micelle nanoparticle	[11,31]
Doxil	PEG-liposome +DOX	80–90	PEGylated liposome nanoparticle with a more extended time of circulation, less toxicity, prevents phagocytosis	[11,31]
Genexol-PM	PEG- poly(l-lactic acid (PLA) micelle+paclitaxel	20–50	Micelle nanoparticle	[11,31]
Abraxane	Albumin+paclitaxel	120 (May dissolve upon exposure to blood)	Albumin nanoparticle	[11,32]
XYOTAX	Poly-l-glutamic acid (PG) + paclitaxel	Not reported	Polymer nanoparticle	[11,33]
LE-SN-38	Liposome+SN-38	Not reported	Liposome nanoparticle	[11,34]
CT-2106	PG+campothecin	Not reported	Polymer nanoparticle	[11,35]
IT-101	Cyclodextrin-containing polymer+campothecin	30–40	Polymer nanoparticle with extended circulation times	[11,36]
CCN	Candesartan cilexetil loaded nanoemulsion	35.5 ± 5.9	Nanoemulsion formulation with increased aqueous solubility	[37]
NCS-DOX	Nanocapsules with oily selol core and a shell of poly(methyl vinyl ether-co-maleic anhydride) + DOX	170	Poly(methyl vinyl ether-co-maleic anhydride) nanocapsules facilitate the co-delivery of drugs	[38]
NCI/NCa	(DNase)-degradable DNA nanoclew embedded with an acid-responsive DNase I nanocapsule (NCa) + DOX	150–180	DNA-based nanoparticle	[39]
MWCNTs/DOX/TC	TAT Chitosan functionalized multi walled carbon nanotube (MWCNT) + DOX	200–300	Multiwalled carbon nanotube-based nanosystem	[40]

In order to circumvent these barriers, researchers have taken advantage of conjugating tumor-targeting ligands to the nanoparticle surface resulting in “active targeting” (Figure 2B) [7,41]. Binding of ligand to exposed cell surface results in “receptor-mediated endocytosis”. However, receptor density plays a pivotal role in such targeting. Commonly used targeting agents are proteins (antibody and its fragments), ligands of the up-regulated receptors (peptides, carbohydrates and other small molecules) and nucleic acid aptamers [11]. Nanotherapeutics used for active targeting under clinical trial are mentioned in Tables 2 and 11. Peptides have been widely used as receptor targeting moieties for active targeting. Table 3 enlists an elaborate list of peptide-based targeting ligands which have already been reported by Gray and Brown [42].

**Table 2:** Antibody and peptide-based nanotherapeutics used for active targeting in the clinical trial

Name	Targeting agent	Therapeutic agent	Status	Comments	Ref.
Gemtuzumab ozogamicin (Mylotarg)	Humanized anti-CD33 antibody	Calicheamicin	FDA approved	Antibody-drug conjugate (ADC)	[11,43]
Denileukin diftitox (Ontak)	Interleukin 2 (IL-2)	Diphtheria toxin fragment	FDA approved	Recombinant fusion protein of IL-2 attached to diphtheria toxin fragments	[11,44]
Ibritumomab tiuxetan (Zevalin)	Mouse anti-CD20 antibody	^90^Yttrium	FDA approved	Antibody–radioactive element conjugate	[11,45]
Tositumomab (Bexxar)	Mouse anti-CD20 antibody	^131^Iodine	FDA approved	Antibody–radioactive element conjugate	[11,45]
FCE28069 (PK2)	Galactose	Doxorubicin	Phase I clinical trial (stopped)	A conjugate of HPMA copolymer, Doxorubicin and galactose	[11,46]
MCC-465	F(ab′)2 fragment of human antibody GAH	Doxorubicin	Phase I clinical trial	Immunoliposome-encapsulated Doxorubicin (DXR)	[11,47]
MBP-426	Transferrin	Oxaliplatin	Phase I clinical trial	Liposomal oxaliplatin suspension for injection	[11,48]
SGT-53	Antibody fragment to transferrin receptor	Plasmid DNA with p53 gene	Phase II clinical trial	A complex of cationic liposome encapsulating p53 DNA sequence in a plasmid backbone	[11,49]
CALAA-01	Transferrin	Small interfering RNA	Phase I clinical trial	Polymer-based nanoparticle having human transferrin protein targeting agent	[11,50]
BIND-014	Prostate-specific membrane antigen (PSMA)–targeted peptide (GDHSPFT, SHFSVGS and EVPRLSLLAVFL)	Docetaxel	Phase II clinical trail	PLGA-PEG nanoparticle	[51,52]

**Table 3:** Receptor targeting peptide sequences

Receptors	Cell line	Peptide sequence	Ref.
Met	MDA-MB-231	YLFSVHWPPLKA	[42]
HER2/ErbB2	MDA-MB-231	KCCYSL	[42]
Transferrin	MDA-MB-231, HeLa	THRPPMWSPVWP	[53]
α_v_β_3_	MDA-MB-231, HUVEC	CDCRGDCFC	[42]
EGFR	MDA-MB-468, MDA-MB-231	YHWYGYTPQNVI	[42]
IL-6 receptor	B9	LSLITRL	[42]
Somatostatin receptor Type 2 (SSTR2)	Breast, ovarian and cervical cancer cell lines	fc[CFwKTC]T(ol) (Octreotide) (f : ^D^Phenylalanine, w : ^D^Tryptophan, c : cyclic)	[54,55]
Ghrelin receptor (GnRH-R)	Breast, lung, ovarian and prostate cancer cell lines	pGlu-HWSYkLRPG-NH_2_ (pGlu : Pyroglutamic acid, k : ^D^Lysine)	[54]
Bombesin/Bn receptors	Prostate, breast, pancreas and small cell lung cancer cell line	yQWAV-βAla-HF-Nle-NH_2_ (y : ^D^Tyrosine, Nle : Norleucine, βAla : β-Alanine)	[54,56]
Vasoactive intestinal peptide receptors (VIP receptors)	Breast, colon and endocrine cancer tumor cells	HSDAVFTDNYTRLRKQMAVKKYLNSILN-NH_2_	[54]
Neurotensin receptor 1 (NTSR1)	Breast, colon and pancreatic cancer cells	RRPYIL	[54,57]
CCK2R	Liver, thyroid and pancreatic cancer cells	eAYGWMDF-NH_2_ (e : ^D^Glutamic acid)	[54]
MC_1_R	Melanoma cells	Ac-Nle-DHfRWGK-NH_2_ (Ac : Acetyl, f : ^D^Phenylalanine)	[54,58]
Human Y1 receptor (hY_1_R)	Ewing sarcoma and breast cancer cell lines	YPSKPDFPGEDAPAEDLARYYSALRHYINLITRPRY-NH_2_	[54]
N-cadherin	HUVEC	SWTLYTPSGQSK	[42]
Carbonic anhydrase IX	Renal cell carcinoma cell lines	YNTNHVPLSPKY	[42,59]

**Figure 3:**
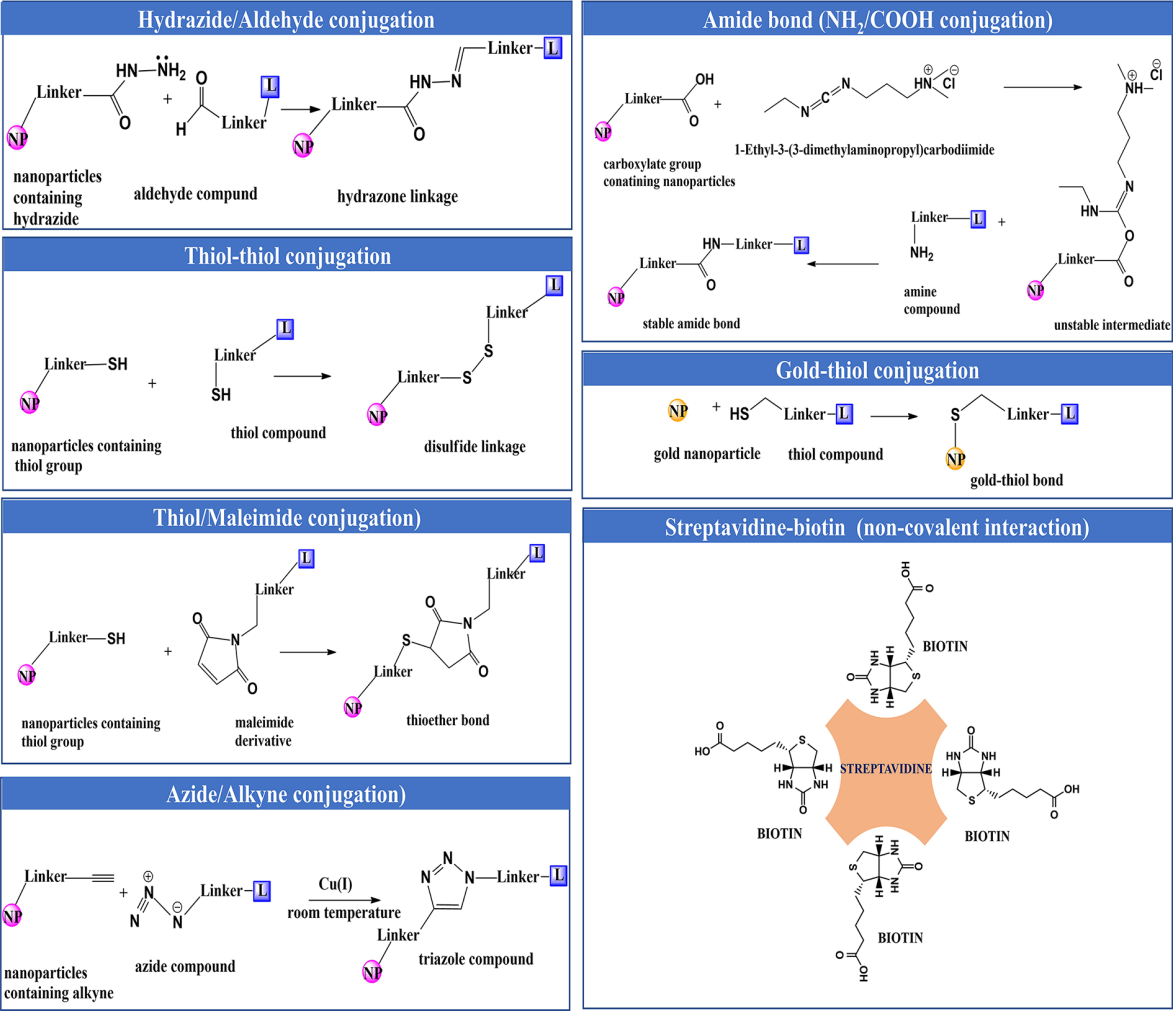
Different types of linkages formed due to various chemical conjugation reactions for active targeting Stability of the linkages under physiological conditions (pH 7.4): Hydrazide-aldehyde conjugation (acid-labile), amide bond (stable), thiol-thiol conjugation (cleaved under reducing condition), gold-thiol conjugation (stable), thiol/maleimide conjugation (stable), azide/alkyne conjugation (stable) and streptavidin-biotin conjugation (stable).

Conjugation of ligands with the nanoparticles is mediated via covalent or non-covalent chemical conjugation. However, non-covalent conjugation often leads to weak bonding making it less efficacious. Covalent coupling is commonly achieved with the conjugations of the following groups (1) hydrazide-aldehyde, (2) carboxylic acid-primary amine, (3) thiol-thiol, (4) gold-thiol, (5) maleimide and thiol and (6) azide-alkyne. Different types of linkages formed due to various chemical conjugation reactions are described in Figure 3 [60,61].

**Figure 4:**
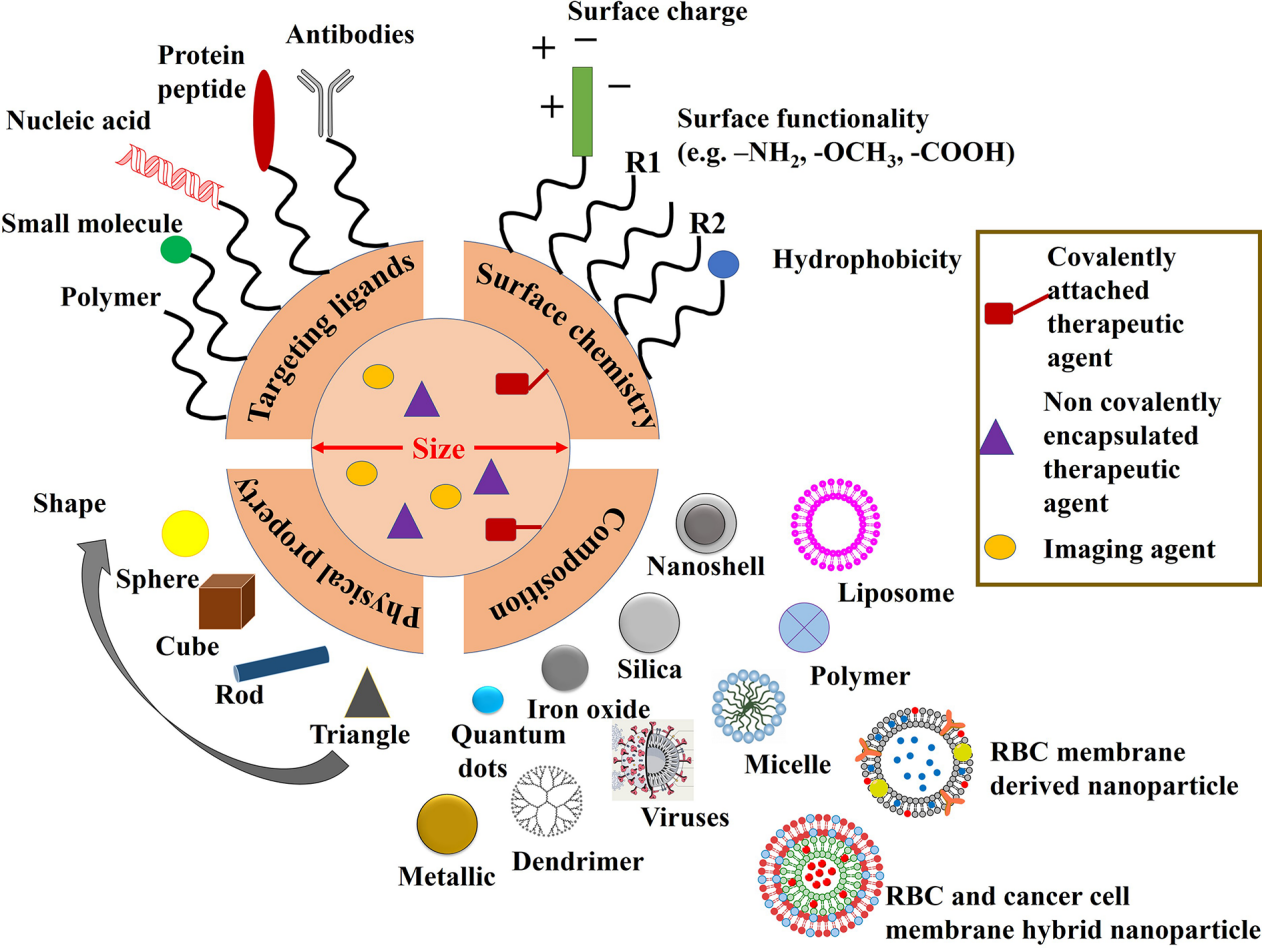
Structural components of multifunctional cancer nanomedicine Commonly used therapeutic agents are chemotherapeutic drugs, RNAi therapeutics, and imaging agents include MRI contrast agents, radionuclides, fluorescent probes, etc. Therapeutic agents can either be covalently conjugated or non-covalently encapsulated. Attaching imaging probes with nanoparticles containing therapeutic agents make it a theranostic platform (this image was drawn based on the information provided in Chou et al. 2011 [70], Figure 1).

**Figure 5:**
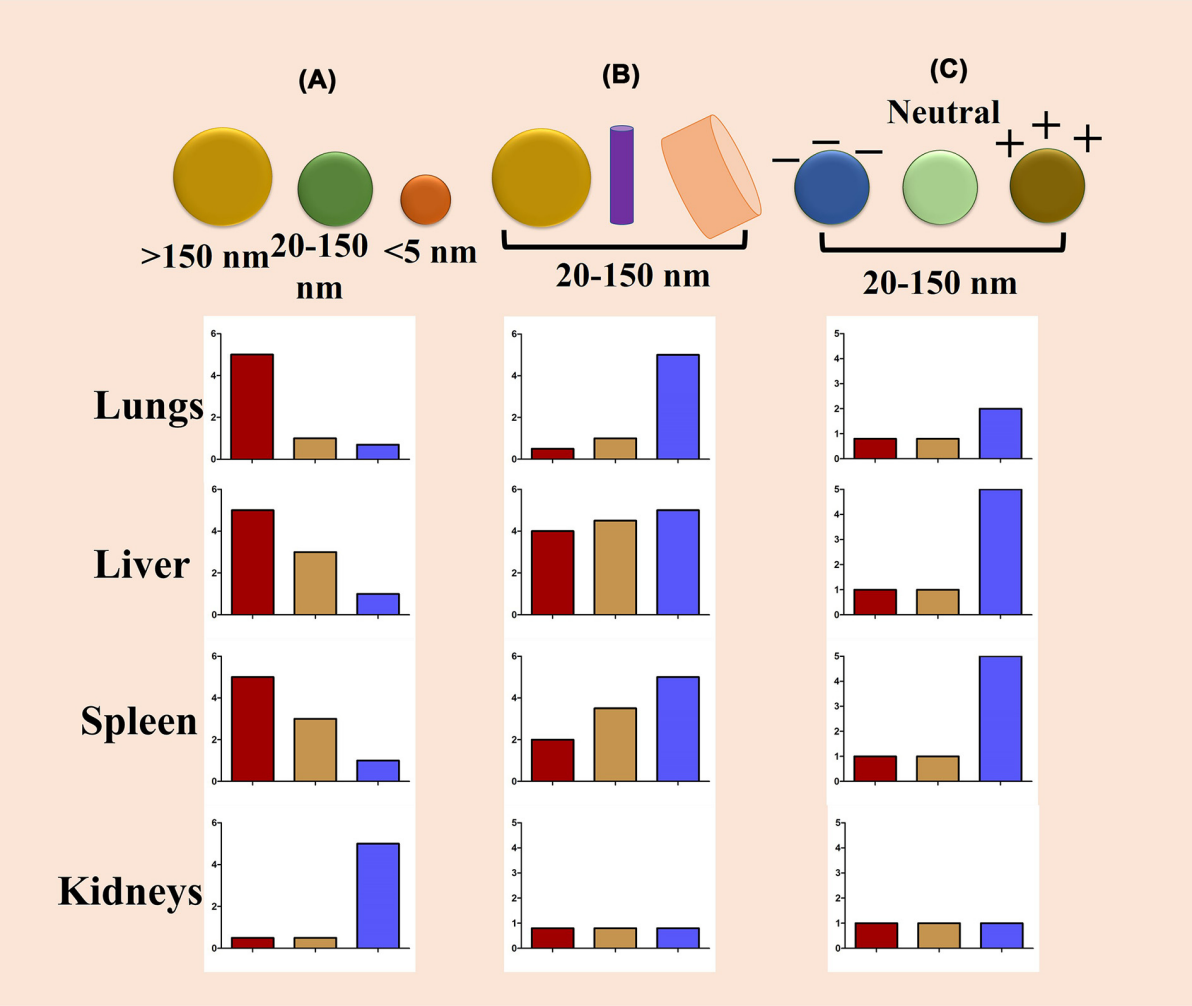
Biodistribution of nanoparticles Nanoparticles having different (**A**) size, (**B**) shape, (**C**) surface charge and their biodistribution in different organs. This figure will guide us for designing organ-specific delivery of nanoparticles (this image was drawn based on the information provided in Blanco et al. 2015 [10], Figure 5).

Size, shape and surface modification also remain essential to achieve effective tumor targeting (Figure 4). In order to specifically target the tumor, nanoparticles must first travel in circulation without being engulfed by macrophages [62]. Nanoparticles having a diameter of ∼5 nm undergo rapid renal clearance, whereas nanoparticles of 50–100 nm diameter mostly accumulate in the liver. Larger particles >2000 nm in diameter tend to accumulate in the spleen. It has been found that nanoparticles having a diameter of 100–200 nm can escape filtration by the liver and spleen (Figure 5) [10]. Researchers observed that nanoparticles of size ranges 30-50 nm diameter showed maximum cellular internalization ability [63]. Studies have also shown that the size of nanoparticles is also dependent on tumor maturity. Reports suggest that with increasing particle size, the area of permeation within tumors become smaller [64]. Gold nanoparticles with 15–45 nm diameter have been found to accumulate in tumors of 0.5-1 cm^3^ volume or above [65]. Recent findings suggest that the shape of the nanoparticles also determines the cellular uptake [66]. Several nanostructures like 2D polygonal shape, 3D polygonal shape, rod, snowflakes, flowers, thorns, hemispheres, cones, filaments, etc., have been designed to study their efficacies [62]. Recent studies have revealed that oblate shape is favoured in circulation [62]. Mitragotri and co-workers have modified a solid polystyrene microparticle into a red blood cell (RBC) shaped particle using layer-by-layer (LbL) self-assembly technique. The nanoparticles were synthesized using PLGA, which can be used as a carrier for drug and imaging agents [67]. Additionally, RBC-derived cell membrane and a hybrid membrane having membrane from RBC and cancer cell line were used for delivering chemotherapeutic drugs and such methodologies can be explored for designing personalized nanomedicine [68,69]. Surface charge of the nanoparticles also plays a significant role in nanoparticle internalization (Figure 4). Current studies have demonstrated that nanoparticles having a size range of 50–100 nm carrying a very slight positive charge favour the penetration in large tumors [11].

**Figure 6:**
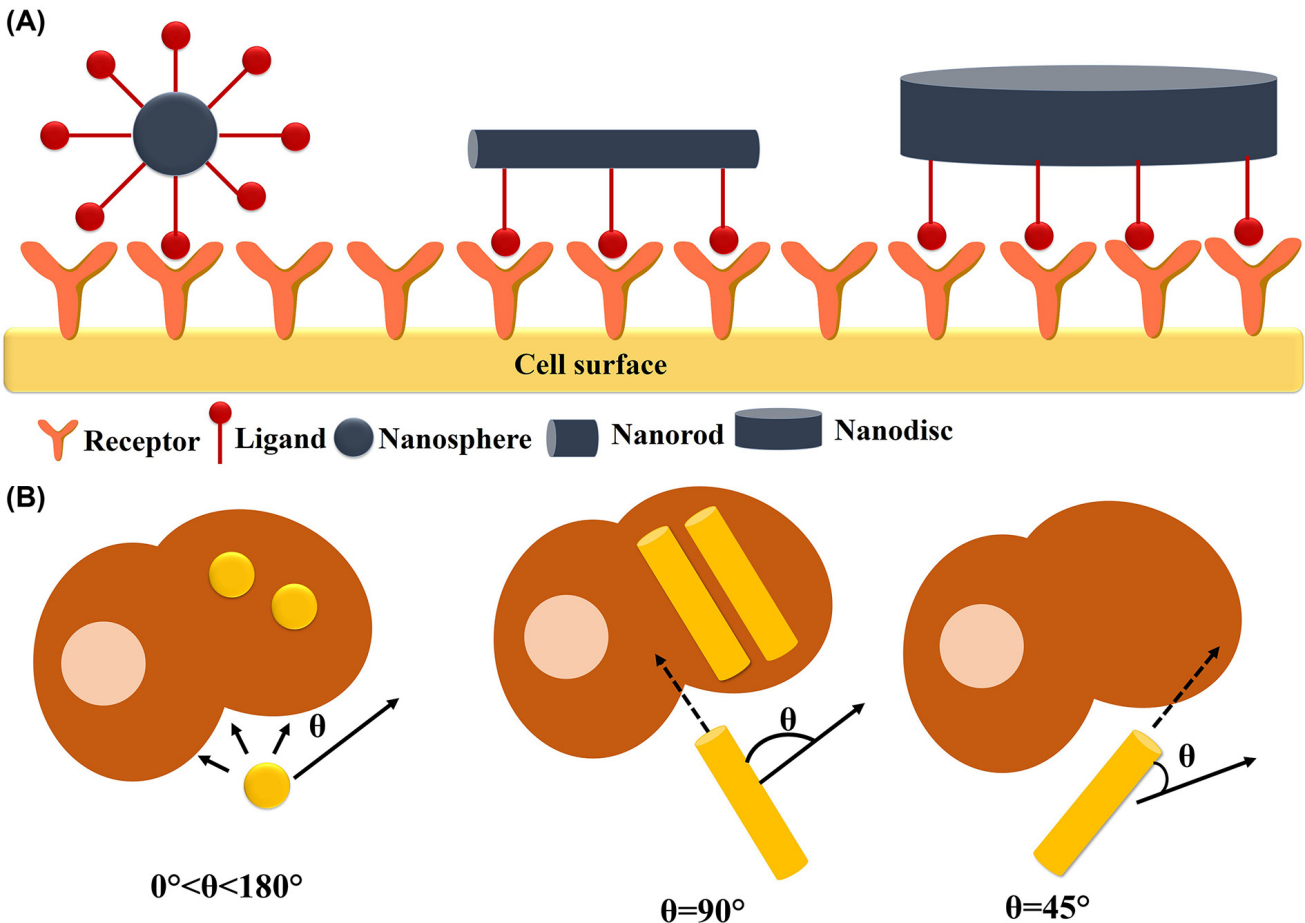
Multivalent interaction of nanoparticles (having different shapes) with cell surface receptors (**A**) Role of the shape of nanoparticles on multivalent interaction with cell surface receptors. Compared with nanospheres, oblong-shaped nanoparticles can form more multivalent interactions, which is required for vascular targeting (this image was drawn on the basis of information provided in Blanco et al., 2015 [10], Figure 3). (**B**) Role of contact angle of nanoparticles in intracellular internalization. Rod-shaped nanoparticles tend to internalize faster when it is present perpendicularly on the cell membrane. Due to the symmetry of the spherical nanoparticles, they do not prefer any specific contact angle (This image was drawn on the basis of information provided in Toy et al., 2014 [62], Figure 2).

**Figure 7:**
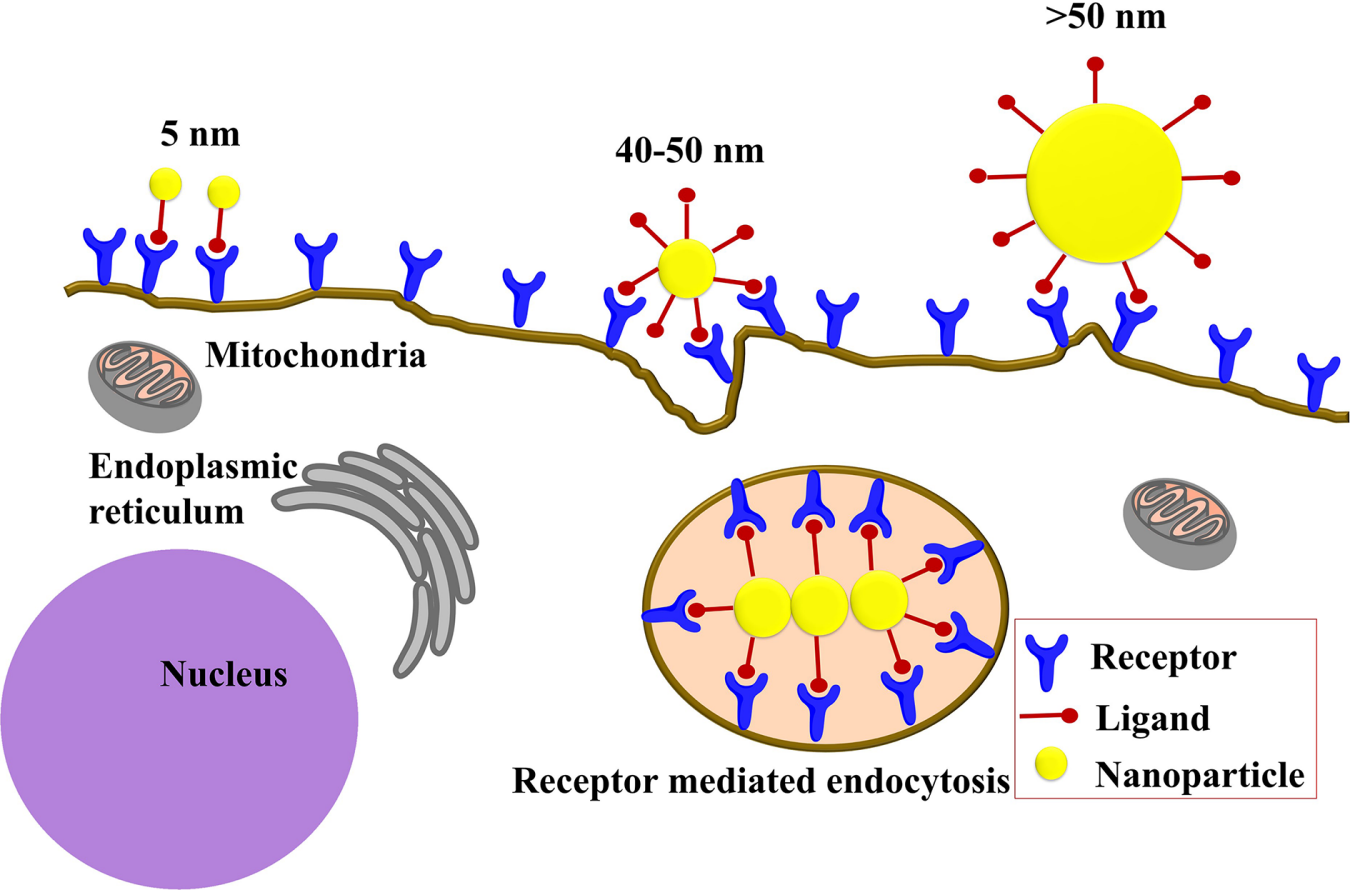
Illustration of size (diameter) dependent nanostructure internalization due to membrane wrapping This figure indicates that 40–50 nm gold nanostructures show optimum cellular uptake activity due to membrane wrapping. Smaller nanoparticles readily dissociate from receptors before being engulfed by the membrane, whereas extremely large nanoparticles fail to cause membrane wrapping (this figure was prepared based on the information provided in Jiang et al., 2008 [71], Figure 3A).

A transient increase in blood pressure during systemic administration also causes increased tumor-specific nanoscale drug delivery. For *in vivo* delivery, near the wall margination is favored that interacts with the tumor vasculature bed. RBCs tend to travel in the middle of the blood flow, creating a “cell-free layer”. Spherical nanoparticles follow the bloodstream whereas, rod-shaped nanoparticles undergo a lateral drift due to variable drag forces and torques. Nanorods show a 7-fold higher accumulation at the vessel lining than nanospheres. Again, discs marginate two times higher than rods. Particles are deposited at a higher rate at the site of the vessel bifurcation [62]. Additionally, the “multivalency” of a nanoparticle can be harnessed to enhance the binding affinity or avidity of ligand to receptor interaction [7]. It has been found that oblong-shaped nanoparticles are more helpful in forming more multivalent interactions compared to spherical nanoparticles (Figure 6) [62]. The cellular internalization process also depends on another factor termed “membrane wrapping time” (Figure 7). Smaller nanoparticles tend to dissociate faster from receptors before being engulfed by the membrane to achieve receptor-mediated endocytosis. Again, extremely large nanoparticles limit the process of membrane wrapping. Chan and co-workers suggested that 40–50 nm nanoparticles remain the critical cut off point for receptor mediated endocytosis [71].

## Controlled drug release

An ideal drug delivery platform should possess the ability to target and control the drug release, which facilitates sustained release of drugs [72]. Drug delivery often renders toxicity and side effects. These hurdles can be overcome by controlled drug release. This feature also leads to a high therapeutic index for the conjugated drug molecule. The binding of the drugs to the nanoparticles is achieved by adsorption, absorption, entrapment (the process of incorporation of a drug into a matrix) and covalent binding. The release of the drug molecules is decided on the basis of their solubility, desorption, diffusion through nanoparticles matrix, degradation of nanoparticle matrix and combination of such phenomena [73,74]. In many of such formulations, a phenomenon called “burst release” is often observed. A large bolus of drug is immediately released before it reaches to a stable profile. Researchers found that low molecular weight drugs are prone to exhibit burst release profile. Burst release may often lead to local or systemic toxicity, short half-life of drugs *in vivo* and shortened release profile [72]. Researchers have often modified the nanoparticles to get over this difficulty. Le and co-workers have used chitosan to modify PLGA nanoparticles carrying paclitaxel and monitored the drug release profile. They found that modification of PLGA nanoparticles with chitosan led to reduced burst release of drug [75]. Again, designed nanoparticles that are prone to transcytosis (the vesicular transport of macromolecules from one side of a cell to the other) possess the potential to reach tumor cells and show efficient anticancer efficacy [76].

Nanotechnology has made advancements in mediating anticancer therapy and imaging at the tumor microenvironment. Several synthetic and natural nanoparticles have been used currently. These include polymeric conjugates and polymeric nanoparticles ((N-(2-hydroxypropyl)methacrylamide (HPMA) copolymers, Poly(lactic co-glycolic acid) (PLGA) copolymers, etc.; liposomes and micelles; synthetic organic nanoparticles such as dendrimers; carbon-based nanostructures such as carbon nanotubes (CNTs) and polyhydroxylated/ PEGylated fullerenes; inorganic nanoparticles of gold, silver, and iron oxide; quantum dots (QDs); viral capsids and ferritin, etc [7]. Liposomes are spherical vesicles comprising of one or more lipid bilayers, especially phospholipids. Liposomes have been widely used as a drug delivery vehicle. These increase the drug’s efficacy and therapeutic index by protecting the drug from the external environment. Dendrimers are radially symmetric branched polymeric nanoparticles. They possess the properties like poly valency, self-assembly, chemical stability, low toxicity and solubility. Dendrimers have been widely utilized to deliver anticancer drugs. Polymeric nanoparticles contain random or block co-polymers. These are colloidal particles having a size range of 1 to 1000 nm. Drug delivery has been widely performed using polymeric nanoparticles, either by encapsulation or chemical conjugation of drugs [18].

The delivery of chemotherapeutic drugs like cisplatin has been rigorously manipulated with nanotherapy to overcome their toxicity. Sengupta and co-workers have designed a novel cisplatin nanoparticle by harnessing PEG-functionalized poly-isobutylene-maleic acid (PEG-PIMA) co-polymer. Cisplatin is released in a pH-dependent manner and shows improved antitumor efficacy both *in vitro* and *in vivo* with limited nephrotoxicity [77]. They have also engineered another novel polymer glucosamine-functionalized PIMA to complex with platinum at a unique platinum to polymer ratio. Such nanoparticles also exhibit improved efficacy against breast and lung cancer with reduced systemic and nephrotoxicity [78]. Sengupta et al. have also designed cholesterol-tethered platinum II-based supramolecular nanoparticle with increased efficacy and reduced toxicity [79]. Prolonged use of cisplatin leads to nephrotoxicity. In order to overcome this limitation, other next-generation platinum-based drugs have been developed. Carboplatin (*cis*-diamino-(1,1-cyclobutandicarboxylate)platinum(II)) has been used recently, but it shows a cross-resistance with cisplatin. Oxaliplatin (*cis*-[(1*R*,2*R*)-1,2-cyclohexanediamine-*N*,*N*′]-oxalatoplatinum (II)) does not show such cross-resistance with cisplatin and is also highly soluble in water. Moreover, trans-1,2-diaminocyclohexane (DACH) ring of oxaliplatin adduct fills the major groove of DNA more efficiently than cisplatin. Scientists have derivatized the monomeric units of a PIMA copolymer with glucosamine, which chelates DACH platinum (II) and releases DACH-platinum in a sustained pH-dependent manner with reduced systemic toxicity and minimal kidney accumulation [80]. Kulkarni et al. also designed a computational algorithm to develop nanoscale supramolecular structures for cancer treatment [81].

Nanotechnologies have also been currently used for genetic treatments by nanoparticle-mediated delivery of RNAi therapeutics [10]. The primary challenge of RNAi-based therapeutics for its successful translation to clinics is the instability of RNA molecules, their rapid degradation in presence of nuclease and poor cellular uptake because of its highly anionic nature [82]. Genetic material, such as antisense oligonucleotides, mRNAs and siRNAs, and in the specific case of plasmid DNA have been used to achieve gene therapy via nanoparticles.

Peptides have been used for targeted delivery of diagnostics and chemotherapeutic agents for anticancer therapy. Peptides remain advantageous over other nanotherapeutics in terms of their self-assembling property, easy synthesis, structural manipulation to achieve protease stability, functionalization property, conjugation to the cell surface receptors and maximum therapeutic efficacy of cargo. Peptides show minimal toxicity, improved biodegradability, rapid renal clearance, and remain stable at physiological conditions. Anticancer agents like paclitaxel/docetaxel, doxorubicin, curcumin, fluorouracil have been successfully loaded on to self-assembled peptides and evaluated for their preclinical and clinical status [83]. Peptides have also been used as a potential molecular transporter based nanosystem to deliver RNAi therapeutics like siRNA [84–86].

Table 4 enlists cancer therapeutics that either have received FDA approval or are currently undergoing clinical evaluation [87].

**Table 4:** Nanomaterials in clinical use

Nanomaterial	Trade name	Composition	Application	Manufacturer	Current status	Adverse effects
**Metallic**						
Iron oxide	NanoTherm	Iron oxide NP conjugate with surface ligand aminosilane	Prostate cancer	MagForce	Phase 2b clinical trial	Acute urinary retention
	Feraheme®;	Iron oxide nanoparticles (coated with polyglucose sorbitol carboxymethylether).	Imaging agent for triple-negative breast cancer, head and neck cancer, nonsmall cell lung cancer etc.	AMAG Pharmaceuticals, Inc.	Phase 3 clinical trial	Constipation, fluid retention in the legs, feet, arms or hands, headache, nausea
Gold	Aurimmune	Tumor necrosis factor (TNF)-gold nanoparticle	Cancer therapy (various cancer types)	CytImmune Sciences	Phase 1 clinical trial	Fever
	Aurolase	Silica-gold nanoshells coated with PEG	Thermal ablation of solid tumors: head/neck cancer, primary and/or metastatic lung tumors	Nanospectra	Pilot study	Inflammation
Nanoshells	Auroshell	Gold metal shell and a non-conducting silica core	Cancer therapy (head and neck)	Nanospectra Biosciences	Phase 1 clinical trial	Under investigation
**Organic**						
Protein	Abraxane	Albumin-bound Paclitaxel for Injectable Suspension	Cancer therapy (breast)	Abraxis Bioscience	FDA approved	Cytopenia
Liposome	Doxil/Caelyx	Liposomal Doxorubicin	Cancer therapy	Ortho Biotech	FDA approved	Hand-foot syndrome, stomatitis
Polymer	Oncaspar	Pegylated form of L-asparaginase	Cancer therapy (acute lymphoblastic leukemia)	Rhône-Poulenc Rorer	Phase 2 clinical trial	Urticaria, rash
	CALAA-01	Formulation of siRNA that consists of a CD-polycation, adamantane (AD)–PEG (MW of 5000) conjugate and AD-PEG-transferrin as the targeting ligand,	Cancer therapy (various cancer types)	Calando	Phase 2 clinical trial	Mild renal toxicity
Micelle	Genexol-PM	Paclitaxel-methoxy polyethylene glycols (mPEG)-Poly(D,L-Lactic Acid) (PDLLA) conjugate	Cancer therapy (Various cancer types)	Samyang	Phase 4 clinical trial	Peripheral sensory neuropathy, neutropenia

## Nanoparticle in targeting oncogenic signaling pathways

Signal transduction pathways play a crucial role in cellular functions like survival, growth, differentiation and metabolism. In the case of cancer, such signaling pathways remain altered, leading to uncontrolled proliferation, immortality and tumorigenesis. Researchers have focused on identifying the drug targets to inhibit oncogenic signaling pathways [28]. Classically, humanized antibodies and small molecule inhibitors have been used as potential inhibitors to target such oncogenic pathways. But nanotherapy has helped target cancer cells selectively without causing toxicity to the healthy cells. The following section mentions key signaling pathways responsible for tumorigenesis and the approaches taken to target such pathways.

## Targeting receptor tyrosine kinase (RTK) pathway for developing anticancer therapy

**Figure 8:**
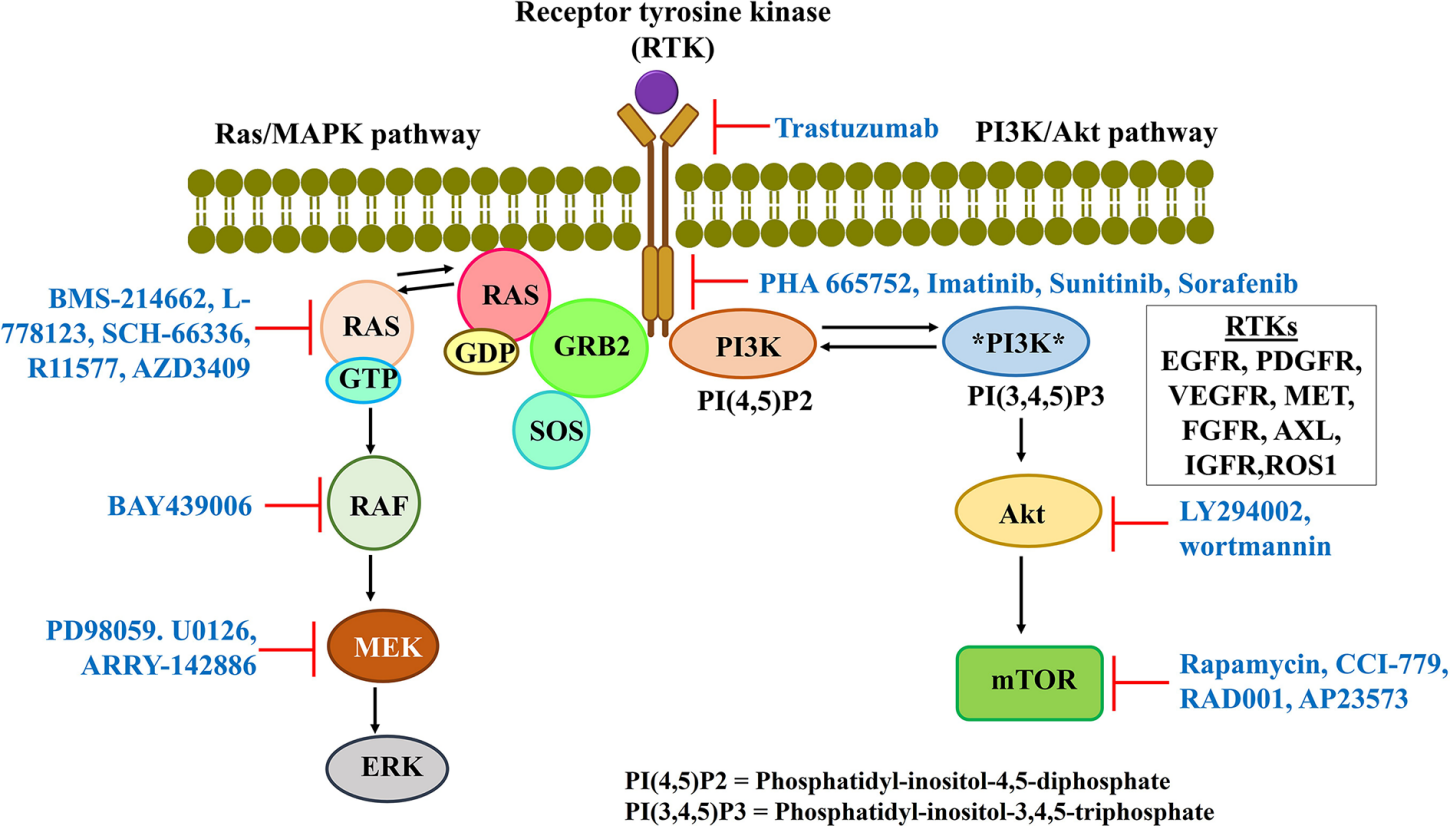
Oncogenic receptor tyrosine kinase pathway with its downstream signaling pathways and small molecule inhibitors targeting different proteins of the network Receptor tyrosine kinase (RTK) plays a significant role in cellular proliferation. MAPK and PI3K-AKT-mTOR pathways are two key downstream pathways of RTK. Targeting these pathways has remained a successful approach to cause the antitumor effect. Small molecule inhibitors have been widely used for targeting different components of such pathways. Several small molecule inhibitors targeting different proteins of RTK signaling pathways are depicted in this diagram.

Receptor tyrosine kinase (RTK) plays a crucial role in major cellular processes like proliferation. Growth factors bind to RTKs, resulting in the dimerization and activation of RTKs. The activation of the intracellular kinase domain of RTKs triggers downstream pathways (Figure 8) [28]. Different types of RTKs have been identified depending on growth factor ligands (e.g., epidermal growth factor receptor [EGFR], vascular endothelial growth factor receptor [VEGFR], fibroblast growth factor receptors [FGFR], platelet-derived growth factor receptor [PDGFR] etc.). In cancer, RTKs are aberrantly activated and their mutations lead to various disorders. Therefore, RTKs and their ligands remain a potential drug target [28]. The inhibitors are mentioned in Figure 8.

Kulkarni et al. found that multi-receptor tyrosine kinase inhibitor (XL184) loaded liposomes mediated nanoscale medicine improves intratumoral concentration, enhances antitumor efficacy and reduces toxicities [88].

Targeting the downstream signaling pathways also act as an alternative strategy. Mitogen-activated protein kinase (MAPK) and phosphatidylinositol-3-kinase PI3K-AKT-mTOR (mammalian target of rapamycin) pathways are two critical downstream pathways [28].

## Targeting mitogen-activated protein kinase (MAPK) signaling for developing anticancer therapy

MAPK pathway comprises a series of proteins like rat sarcoma virus (RAS), rapidly accelerated fibrosarcoma (RAF), mitogen-activated protein kinase kinase (MEK) and extracellular signal-regulated kinase 1/2 (ERK 1/2). This pathway is up-regulated in most cancer types and responsible for abnormal proliferation leading to tumorigenesis. RAS, RAF and MEK gained the most attention as therapeutic targets [28]. The progress in the therapeutic strategy targeting MAPK pathway using small molecule inhibitors is mentioned in Figure 8.

Researchers found that PD98059, a selective MAPK inhibitor, conjugated with hexadentate-poly-D,L-lactic acid-co-glycolic acid polymer potentiate the anticancer efficacy of cisplatin chemotherapy [89].

## Targeting phosphoinositide 3-kinase (PI3K) pathway for developing anticancer therapy

Phosphoinositide 3-kinase (PI3K) pathway is responsible for cellular processes like proliferation, growth, survival and apoptosis. This pathway is mutated in 30% of all human tumors. Activated RTKs set off several downstream signaling cascades, especially protein kinase B (Akt) and mammalian target of rapamycin (mTOR), responsible for various cellular functions [28]. The small molecule inhibitors targeting this oncogenic signaling pathway are mentioned in Figure 8.

Utilizing the nanotechnology, Harfouche et al. found that encapsulating LY294002, a PI3 kinase inhibitor into biodegradable PLGA nanoparticle causes successful inhibition of Akt phosphorylation resulting in the inhibition of proliferation [90]. Sengupta and co-workers have rationally conjugated PI3K inhibitors (PI103 and PI828) using a cholesterol-based derivative, enabling supramolecular nano assembly with L-α-phosphatidylcholine and DSPE-PEG [1,2-distearoyl-sn-glycero-3-phosphoethanolamine-N-[amino(polythylene glycol)] to achieve increased antitumor efficacy [91]. They have also suggested that a rational combination of cis-platinum nanoparticles and a PI3K-targeted therapeutic remains a potential therapy for breast cancer [92]. Free nanoparticles or nanoparticles carrying different therapeutic agents like oligonucleotides, cytotoxic drugs, antibodies, etc., have been used to target different oncogenic pathways (Table 5).

**Table 5:** Nanoparticles used to target signaling pathways

Nanocarriers	Materials/drugs	Cell line/animal model	Effects	Ref.
Gelatin nanoparticles (D-NPs: gelatin nanoparticles loaded with NF-kB inhibiting decoy oligodeoxynucleotides)	NF-kB inhibiting decoy oligodeoxynucleotides (Decoy oligonucleotides (decoy ONs) epitomize an ideal pharmacological tool to selectively block NF-κB activation.)	Kupffer cells	Inhibition of NF-κB activation by D-NPs in kupffer cells (KC) D-NPs inhibit the nuclear translocation of p65, a common subunit of NF-kB. Improve survival and reduction in liver damage	[93]
Folate-linked lipid-based nanoparticles	NF-kB decoy	RAW264.7	The NF-κB decoy shows an inhibitory effect in cytoplasm (inhibition of NF-kB translocation into nucleus of LPS-activated macrophages) Inhibit particular matter (size ≤2.5µm) (PM2.5) induced neuroinflammation	[94]
Fisetin nanoparticles (FN) (Fisetin, a natural flavonoid)	No cargo	C57BL/6 mice primary astrocytes	Restrict PM2.5 exposure-induced NF-κB signaling activation. Decrease PM2.5-induced astrocytes activation. Reduce pro-inflammatory cytokines IL-1β and TNF-α	[95]
mPEG-PLGA nanoparticles	Benzoylaconitine	RBCs, RAW264.7 cells	Inhibit the expression of NF-κB p65 Inhibition of NF-κB signaling to reduce inflammation	[96]
Silica nanoparticles (MSN-mesoporous silica nanoparticles)	NF-κB p65 antibody (p65, also known as RelA)	Balb/c mice	Translocation and cell signaling transduction (the nanoparticle binds to p65, forming a complex, thereby inhibiting the entry of p65 into nucleus)	[97]
Nano-Selenium (Nano-Se)	No cargo	Albino wistar rats	Exhibit negative NF-κB immune expression. Reduces pancreatic injury and improves pancreatic functions	[98]
Astragalus polysaccharide nanoparticles (Astragalus polysaccharide (APS) is a water-soluble heteropolysaccharide with bioactive effects, *A. Membranaceus* stems or dried roots derivative)	No cargo	H9c2 cells/ C57BL/6 mice	Inhibition of TLR4/ NF-κB pathway activation Decrease the secretion of proinflammatory cytokine	[99]
Niosome nanoparticle (vesicles composed of biodegradable non-ionic surfactants, which is an alternative to liposomes)	Curcumin	Human glioblastoma stem-like cells (GSCs)	Reduce the mRNA expressions of NF-κB and IL-6 and increase the expression of Bcl2 Induce cell cycle arrest, ROS generation and apoptosis Anti-tumor effect	[100]
ABI-009 (albumin-bound-rapamycin nanoparticle)	Rapamycin	Gastroenteropancreatic or lung neuroendocrine tumors patients	mTOR pathway inhibitor targeting cells with TSC1/TSC2 genes mutations	[101]
Mesoporous silica nanoparticles	γ-secretase inhibitors (GSIs)	FVB/N adult mice	MSNPs carrying GSIs used block Notch signaling	[102]
Gold nanoparticles (AuNPs)	No cargo	C3H/HeN mice, NOD-SCID mice	AuNPs could deactivate TGF- β1(cys-rich protein) by directly binding to the cysteine buried inside the protein through covalent bonds (S–Au bonds ≈ 40 kcal mol^-1^), disrupting the disulfide bond in the protein, thus destroying the structure and unfolding the protein.	[103]
Anthothecol-encapsulated PLGA-nanoparticles (Antho-NPs)	Anthothecol (Anthothecol, is a limonoid isolated from a plant named *Khaya anthotheca* (Meliaceae), which acts as an antimalarial compound)	AsPC-1,PANC-1 and Mia-Paca-2 cell	Antho-NPs are found to actively inhibit the expression of Gli, Patched1, Bcl-2 and CyclinD1 in pancreatic CSCs. Antho-NPs suppresses cell motility, migration and invasion by up-regulating E-cadherin and inhibiting N-cadherin and Zeb1	[104]

Another approach to target oncogenic signaling pathways is mediated by siRNA delivery, a component of gene therapy. Peptides have also been used as a molecular transporter-based nano-system to deliver siRNA targeting signaling pathways [86,105,106,107]. This is discussed in the later section of this review.

## Nanoparticle in tumor angiogenesis

Angiogenesis is a phenomenon of the formation of the new blood capillaries out of pre-existing blood vessels. This phenomenon is essential for wound healing [108–111]. Cancer metastasis is also positively correlated with angiogenesis [112]. Therefore, blocking angiogenesis is a practical approach to inhibit cancer progression. Commonly available anti-angiogenic therapy harbors any of these two strategies: (1) damaging the existing blood vessels or (2) preventing the formation of new blood capillaries [113]. Anti-angiogenic therapy is different from commonly used tumor-targeted chemotherapy. It selectively targets the tumor-associated vasculature instead of the tumor cells, shows increased bioavailability for systemically administered drugs compared with tumor-targeted therapy and requires a low administration dose, leading to lesser systemic toxicity [113,114].

In spite of these advantages, chemotherapy-mediated anti-angiogenic therapy suffers from certain limitations. The tumor vasculature remains unreachable to most of the anti-angiogenic inhibitors. Nanotechnology has become an advanced and effective method to address this problem. Nanoparticle-mediated delivery of therapeutics has been achieved following both passive and active targeting [113]. Researchers have shown that conjugating cytotoxic drugs or angiogenic inhibitors with nanoparticles prefer to home at the tumor site following the EPR effect [115,116]. Active targeting has also been effective in targeting angiogenesis. Anti-angiogenic therapy harbors targeting VEGFR, α_v_β_3_ integrins and other angiogenic factors. Synthetic peptides having the recognition site for integrins (cyclic Arginine-Glycine-Aspartic acid [cRGD]) have been widely used for targeted nanotherapy [113]. A plethora of nano-vectors have been reported by researchers for nanotechnology-based anti-angiogenic therapy, including polymeric nanoparticles, liposomes and micelles, dendrimers, carbon nanostructures, inorganic nanoparticles (e.g., gold, silver and iron oxide), etc. Recent nanotechnology-based anti-angiogenic therapies also use a gene silencing-based approach using therapeutic genes or siRNA [113].

A few reports on nanotechnology-based anti-angiogenic therapy are provided in Table 6.

**Table 6:** Nanoparticles used for anti-angiogenic therapy

Nanoparticle	Cargo	Used against	Comments	Ref.
**Polymer**				
HPMA	TNP-470 (Caplostatin)	Human melanoma, lung carcinoma	Prevents crossing of blood–brain barrier (BBB), limiting neurotoxicity	[117]
	Aminobisphosphonate drug alendronate (Fosamax) and paclitaxel/TNP-470	Osteosarcoma	Inhibits bone metastasis	[118]
	Radionuclidelabeled, cyclized RGD peptide	Solid tumors	Used for diagnosis and therapeutic application	[119]
PLGA	LY294002 (PI3K pathway inhibitor)	Zebrafish melanoma, breast adenocarcinoma	Shows anti-angiogenic effect	[90]
PLGA nanoparticle encapsulated within PEG linked lipid envelop	Doxorubicin (covalently attached to inner PLGA core) and anti-angiogenic agent (combretastatin)	Melanoma	Termed as “nanocell,” shows an improved therapeutic index with reduced toxicity	[115]
PEGylated polyethyleneimine (PEI) consisting RGD peptide PEGylated polyethyleneimine (PEI) consisting RGD peptide	sFlt-1 gene	Colon carcinoma	Blocks VEGF binding to membrane-bound Flt-1 receptor and inhibits proliferation	[120]
	VEGFR-2 targeting siRNA	Mouse neuroblastoma	Inhibits angiogenesis	[121]
**Polysaccharides and dendrimers**				
Chitosan coated poly-isohexylcyanoacrylate nanoparticle	Anti-RhoA siRNA	Breast cancer mouse xenograft model	Inhibits tumor growth and metastasis	[122]
Boronated polyamidoamine dendrimer	VEGF_121_	Colon carcinoma in mice	Shows anti-angiogenic effect	[123]
**Lipid-based nanoparticles (liposome and micelle)**				
Monomethoxy-polyethyleneglycolpolylactic acid copolymer	TNP-470	Mouse melanoma	Forms a micelle termed “Lodamine” Inhibits angiogenesis	[124]
Poly(ε-caprolactone)-polyethyleneglycol (PCL-PEG)	Cyclic RGD pentapeptide (conjugated) and Doxorubicin (loaded)	Kaposi’s sarcoma	Forms a nanopolymeric micelle Shows antitumor activity	[125]
Ala-Pro-Arg-Pro-Gly (APRPG) peptide (for active targeting), PEG and hydrophobic anchor distearoylphosphatidylethanolamine (DSPE)	Adriamycin	Colon carcinoma	Shows antiangiogenic effect	[126]
Neutral liposome	Protein activated receptor-1 (PAR-1) siRNA	Mouse melanoma	Inhibits metastasis	[127]
**Carbon nanostructures**				
Fullerenols	Doxorubicin	Mouse melanoma	Shows anti-angiogenic effect	119
**Inorganic nanoparticles**				
Dextran coated iron oxide nanoparticles	Radiolabeled anti-VEGF monoclonal antibody	Liver cancer in mice	Destruction of tumor with increased imaging resolution	[128]
Folate receptor targeted superparamagnetic iron oxide nanoparticle	Doxorubicin	Liver cancer	Does not show systemic toxicity	[129]
PEGylated gold nanoparticle	Doxorubicin	Liver cancer in mice	Shows antitumor activity	[130]

## Nanoparticle-mediated gene therapy for cancer

Gene therapy is the modulation of gene expression towards treating a disease by cellular delivery of therapeutic nucleic acid. It holds unique promise in alteration of specific tumor genes functioning via gene addition, gene correction or gene knockdown [16]. Several approaches of cancer gene therapy include (1) suicide gene therapy: introduction of an enzyme expressing transgene into the cell that converts inactive prodrug into cytotoxic metabolite for host cells, (2) gene silencing: suppression of gene expression by RNAi techniques like siRNA, shRNA, antisense oligonucleotide, miRNA, etc., and (3) DNA vaccine: introduction of specific antigen encoding plasmid DNA into the cell to induce immune response.

Gene therapy shows the potential to deal undruggable targets to treat cancer as compared with conventional treatment by targeting cancer associated genes. Gene therapy can provide a solution to low bioavailability, reducing immune system based recognition and delivery of the gene regulators [131]. The fundamental challenge in the engineering of gene therapy is the development of clinically safe and effective delivery vectors. In clinics, both viral and non-viral mode of delivery is being used for systemic gene delivery [132]. The viral delivery systems are associated with various safety concerns as well as limited payload capacity and difficulty in large-scale production [16]. These factors led to the development of interest toward non-viral synthetic vectors for gene therapy. The non-viral vectors are advantageous in providing higher safety profile, low cost, large scale manufacturing potential, stability and higher payload [132]. Nanoparticle and nanoscale gene delivery vectors have emerged as efficient candidates for intracellular or systemic gene delivery.

Nanoparticle exploitation for gene delivery can be categorized into four groups : (1) lipid-based nanoparticles, (2) polymer-based nanoparticle, (3) peptide-based nanoscale material and (4) inorganic nanoparticles.

## Lipid-based nanoparticles

Lipid-based nanoparticles are the most widely used non-viral gene delivery vehicle [132,133]. Cationic liposomes are amphiphilic molecules which are made up of cationic polar head group, a hydrophobic domain and a linker connecting the polar head group with the non-polar tail. This cationic liposomes are routinely used for gene delivery [134]. Incorporation of longer lipidic chain (having around 18 methylene group, which can span the whole membrane) having unsaturation and small polar head facilitates the formation of conical shaped lipid in anionic membrane environment, which promotes the hexagonal phase transition of the lipid bilayer from the lamellar phase [135]. Hexagonal phase of the lipid bilayer is more fusogenic than lamellar phase. This transition of lamellar phase to hexagonal phase of lipid bilayer leads to cellular internalization and endosomal release of the internalized lipid-based nanoparticle [135]. Lipid nanoparticle containing ionizable lipids are designed in a way that it gets protonated at endosomal pH range (4.5–6.5). The protonated cationic lipid interacts with the anionic lipid of the endosomal membrane, leading to the transition of the lamellar phase to the hexagonal phase of lipid bilayer. Several liposomal nanoformulations have been in clinical development like DOTAP-cholesterol, GAP-DMORIE-DPyPE, etc. The FDA approval of Alnylam’s patisiran in 2018, is the first ever drug to successfully harness RNA interference to silence disease associated gene expression was a key milestone. Further, FDA approved lipid based nanoparticles like givosiran, lumasiran established a benchmark for lipid nanoparticle based drugs. Hou et al. have discussed in detail about the use of lipid based nanoparticles for gene delivery, their drawback and engineering principles for engineering next-generation improved lipid based nanoparticles [136].

## Polymer-based nanoparticles

The chemical diversity and functionalization potential of cationic polymer based nanoparticle makes it an attractive class of non-viral gene delivery vehicle. Polymers are considered as inert but certain modification by biologically active agents and counterions like spermidine or cell penetrating peptides improves the surface functionalization, nucleic acid loading and particle transfection [16] PLGA nanoparticle is capable of delivering nucleic acid with minimal toxicity but exhibit low transfection efficiency [16]. PLGA nanoparticles are surface functionalization with cell targeting or cell penetrating peptides to improve nucleic acid loading and cell penetrability. The condensation of negative phosphate bonds of nucleic acid with the cationic polymers into polyplexes protects nucleic acid from degradation during circulation [16].

Polyethylenimine (PEI) and its variants are among the most studied polymeric materials for gene delivery. The presence of nitrogen at every third position of the polymer increases the charge density and reduces the pH [132,137]. Various polymer-based nanoparticles such as dendrimers, polyion complex micelles (PICs), cyclodextrin, etc., have also been explored greatly in gene therapy. The polymeric nanoparticle system owing to their facile synthesis and flexible properties proves to be a new promising material for developing non-viral gene delivery system. The cationic polymer can be conjugated with the negatively charged genetic material via electrostatic attraction at physiological pH and facilitating gene delivery. Ekladious et al. have discussed in detail about the rational designing, physicochemical characteristics and advancements in different classes of polymer based delivery vehicle and their application in different fields including gene therapy [138].

## Peptide-based nanoscale material

Cell penetrating peptides (CPP) are a class of peptides facilitating the cellular internalization of nucleic acid based therapeutics either by covalent or non-covalent conjugation. The cellular uptake of the CPP peptide depends upon their sequence, structure, concentration and cell lines used for study [139]. The cellular internalization of cell penetrating peptides follow either by one or by the combination of the following mechanisms: (1) creating a transient pore in cell membrane, (2) endocytic uptake and (3) receptor mediated uptake. Depending upon the sequence, the CPP can be of cationic CPP or amphiphilic CPP.

The cationic CPPs are arginine or lysine rich short peptides. Arginine rich sequences are found to show enhanced cellular uptake. The arginine can form a more efficient bidentate hydrogen bond than the monodentate hydrogen bond formed by lysine with the anionic moieties like phosphate (PO_4_^3−^), carbonate (CO_3_^2−^) and sulfate (SO_4_^2−^) on the cell surface [140]. These interactions facilitate the cellular internalization of the arginine or lysine residue enriched cationic peptides.

Amphiphilic peptides show enhanced cellular uptake by the formation of lipid rafts in the cell membrane. The cationic part of the CPP interacts with the negatively charged therapeutic nucleic acid and the hydrophobic part facilitates the cellular internalization of the peptide by interacting with the lipid bilayer [141,142].

N-Methylpurine DNA Glycosylase or MPG (GALFLGFLGAAGSTMGAWSQPKKKRKV) peptide is a great example of amphiphilic CPP. The hydrophobic stretch of this amphiphilic CPP (underlined) adopts a transient β-sheet structure creating a temporary channel in the cell membrane allowing the peptide–nucleic acid complex to internalize [143,144]. Amphiphilic α-helical peptide like penetratin (RQIKIWFQNRRMKWKK) generally remains unstructured in an aqueous solution but tends to adopt an α-helical conformation while interacting with cell membrane [84]. Majority of CPP internalizes via endosomal pathway and are accumulated inside endosome. Two strategies employed for endosomal escapes are use of (1) conformation changing fusogenic peptides and (2) proton buffering peptides [84]. The conformation changing fusogenic peptides in endosomal pH (4.5–6.5) undergoes a conformational change and ruptures the endosomal membrane by forming an amphiphilic helix [84]. These peptides are rich in histidine and glutamic acid and are sensitive to pH change. pH sensitivity arises due to imidazole group of histidine side chain having p*K*a value of ∼ 6.0 and glutamic acid side chain with p*K*a ∼ 4.3 allowing easy protonation and de-protonation of these amino acids in endosomal environment.

Proton buffering peptides escape the endosomal entrapment by accumulation of proton absorbing peptides inside endosome and disrupting the endosomal membrane by proton sponge effect. Imidazole ring of histidine having a p*K*a value of nearly 6.0 shows high buffering effect by acting as a weak base inside endosome. This causes influx of protons into the endosome and osmotic swelling causing endosomal rupture. Peptides provide advantage as a delivery vehicle due to their biocompatibility, biodegradability and sheer limitless combinations and modifications of amino acid residues inducing the assembly of modular, multiplexed systems [145]. Tarvirdipour et al. have discussed in detail the designing principles and attractive features of peptide based nanoscale materials for gene therapy [145].

## Inorganic nanoparticles

This includes carbon nanotubes, magnetic nanoparticles, calcium phosphate nanoparticles, gold nanoparticles and quantum dots that are commonly used for gene delivery vehicles. The inorganic nanoparticles are resistant to microbial attacks and provide good storage stability [146]. The functionalized single-walled nanotubes are reported to enter premyelocytic leukemia and T-cell very easily, this ability is exploited to deliver nucleic acid into mammalian cells [147]. Water soluble allotrope of carbon (C60) fullerenes modified to aminofullerenes has positive charge on them. This aminofullerenes are reported to have high transfection efficacy of DNA into mammalian cells [148]. Inorganic nanoparticles are reported to have great gene delivery efficacy on surface modification. Chen et al. have discussed in great detail about the use of inorganic nanoparticle as a drug codelivery nanosystem [149]. Table 7 lists out some of the important nanoparticle based gene therapeutic approaches. Advancement in clinical studies of engineered siRNA-loaded nanoparticles has been discussed in Table 8 [150,151].

**Table 7:** Nanoparticle-mediated gene therapy for cancer treatment

Type of gene therapy	Drug	Nanoparticles used	Cancer type/cell line	Effects	Ref.
Suicide gene therapy	Plasmid DNA encoding saporin gene	U11 peptide functionalized lipid-protamine-DNA nanoparticle	Triple negative breast cancer (MDA-MB-231)	Tumor size was found to be significantly reduced in *in vivo* mice model	[152]
Suicide gene therapy	Plasmid DNA encoding Herpes simplex virus thymidine kinase (HSVtk) gene	Poly(beta-amino ester) nanoparticles	Pediatric brain tumors	Increased median survival in *in vivo* mice model	[153]
siRNA-based therapy	c-Myc siRNA	Gold-PEG nanoparticles	Adenocarcinoma	Reduction in tumor size by 80% *in vivo*	[154]
siRNA-based therapy	Akt1 siRNA	Polyethylenimine based nanoparticle	Mouse colon cancer	Reduced tumor growth	[155]
siRNA-based therapy	Polo-like kinase-1 siRNA (siPLK1)	Hyaluronan containing lipid-based (cholesterol, DSPC, Dlin-MC3-DMA, DMG-PEG and DSPE-PEG amine) nanoparticle	Glioblastoma	Increased gene silencing efficiency and higher survival in mice	[156]
siRNA based therapy	Cell division cycle-associated protein 1 (CDCA1) siRNA	Lipid (PEG lipids, PEG-C-DMA lipids, D-Lin-DMA lipids, DSPC and PEG) nanoparticles	Hepatocellular carcinoma	Significant anticancer efficacy	[157]
siRNA-based therapy	Cyclin targeting siRNA	Peptide MPG (ac-GALFLGFLGAAGS TMGAWSQPKKKRKV-Cya) (Cya : Cysteamide)	HS68 fibroblasts, HeLa, PC3, MCF-7 and SCK3-Her2	Block cancer cell proliferation by efficient down-regulation of cyclin B1 levels	[105]
siRNA-based therapy	ERK1/2 silencing siRNA	Peptide c[-Arg-^D^His-Arg- ^D^His-Arg-Lys (Lys(linoleyl)_2_)-Arg- ^D^His-Arg-^D^ His-Arg-Glu-]-Lys(FAM)-NH_2_	Breast cancer (MDA-MB-231)	Down-regulation of Erk1/2 gene level in TNBC cell line MDA-MB-231	[86]
siRNA-based therapy	Raf-1 siRNA	Peptide based Modified block copolymers-poly(ethylene glycol)- poly(ε-caprolactone)-Tat (GRKKRRQRRRG) (MPEG-PCL-Tat)	C6 cells	Cell death in rat glioma cells	[106,158]
siRNA-based therapy	VEGF siRNA	Peptide KALA (WEAKLAKAL AKALAKHLAKALAK ALKACEA)	PC-3 cells	EGF sequence-specific gene inhibition in prostate carcinoma *in vitro*	[107]
siRNA-based therapy	Polo like kinase-1 siRNA	Multi-walled carbon nanotubes with amino functionalization	Human lung carcinoma	Significant regression of tumor volume	[159]
siRNA-based therapy	Cyclin B1 siRNA and survivin siRNA	Calcium phosphate nanoparticles	Non-small cell lung cancer	Significant gene silencing, reduction in cell growth and induction of apoptosis	[160]
miRNA-based therapy	Tumor suppressor miR-31 and oncogenic miR-1323	Cysteamine functionalized gold nanoparticle	Neuroblastoma and ovarian cancer	Increased payload, efficient cellular uptake and reduced toxicity	[161]
miRNA-based therapy	AntimiR-21 and antimiR-10b	uPA peptide conjugated PLGA-b-PEG nanoparticles	Triple negative breast cancer	Reduction in tumor growth by 40%	[162]
shRNA-based therapy	Doxorubicin encapsulated PLGA nanoparticle and Bcl-xL shRNA	Alkyl modified polyethylenimine	Breast cancer cell line MCF-7	Increased apoptosis of tumor cells and enhanced synergistic effect in comparison with only doxorubicin encapsulated PLGA nanoparticle treatment	[163]
shRNA-based therapy	Akt1 shRNA	Folate and chitosan grafted polyethylenimine copolymer	Human lung carcinoma	Enhanced cell transfection and reduced tumorigenesis	[164]
DNA vaccine	Plasmid encoding prostate stem cell antigen	Cationic RALA peptide/pDNA nanoparticles	Prostate cancer	Showed anticancer activity *in vivo*	[165]
mRNA vaccine	mRNA encoding tumor associated antigen gp100 and TRP-2	Lipid based (DOTAP, DODAP, C12-200, cKK-E12, DOPE, DSPC, POPE, DMPC, DOPS, cholesterol, PEG, arachidonic acid, oleic acid, myristic acid) nanoparticle	Melanoma (B16F10)	Promoted enhanced cytotoxic T-cell response and reduced rate of tumor growth	[166]

**Table 8:** Nanocarrier-mediated siRNA delivery systems in clinical trials

Drug	Disease	Target	Nanoparticle	Company	Status
**Phase I**					
CALAA-01	Solid tumors	RRM 2 (RNA recognition motif domain of the Rbfox family protein)	Cyclodextrin/PEG, transferrin	Calando Pharm	Terminated
ALN-VSP02	Solid tumors with liver lesion	VEGF, KSP (kinesin spindle protein)	SNALP (stable nucleic acid-lipid particles)	Alnylam Pharm.	Completed
siRNA–EphA2–DOPC	Advanced cancer	EphA2 (tyrosine kinase)(a key effector of the MEK/ERK/RSK pathway)	Liposome	M.D. Anderson Cancer Center	Not completed yet
**Phase II**					
FANG	Ovarian tumors	FURIN (a protease enzyme)	Liposome	Gradalis, Inc.	Active
Atu027	Advanced or metastatic solid tumors	PKN3 (protein kinase N3)	Liposome	Silence Therapeutics	Completed
siG12D LODER	Pancreatic ductal adenocarcinoma	KRASG12D	Polymer matrix	Silenseed Ltd.	Ongoing
TKM- 080301 or TKM-PLK1	Solid tumors	PLK1	SNALP	Tekmira Pharma	Completed
DCR-MYC	Hepatocellular carcinoma, solid tumors, non-Hodgkins lymphoma, multiple myeloma, pancreatic neuroendocrine tumors	MYC	Systemic/IV infusion	Dicerna Pharmaceuticals	Terminated

## Nanoparticle-mediated combination therapy against cancer

Combination therapy, a treatment modality involving combination of two or more therapeutic agents, has now become the cornerstone of cancer therapy since single drug based monotherapy failed to provide a considerable therapeutic response. Enhanced therapeutic potential, reduced toxicity and prevention of drug resistance mediated by combination therapy have hugely increased the therapeutic response of cancer patients. Combination therapy can be designed in two different ways. First, targeting of different molecular pathways by multiple drugs can lead to delayed cellular adaptation and oncogenic mutations. Second, targeting the same pathway could be helpful in the development of synergistic interaction of multiple drugs with higher efficacy and target selectivity. Synergistic interaction is best described by the Chou-Talalay method where combination index (CI) is less than 1 [167,168]. Boshuizen et al. suggested that a synergistic drug interaction in a combination therapy can be rationally designed by invoking several principles: (1) multiple targeting of a signaling pathway, (2) maximal driver pathway inhibition, (3) targeting agents responsible for signal reactivation, (4) enhanced synthetic lethality, (5) targeting heterogeneous and drug resistant cell populations in a tumor, (6) targeting immune cell function and tumor microenvironment modulation and (7) neoadjuvant therapies [169]. Combination therapy also employs repurposing of drugs that were initially used for diseases other than cancer. This approach saves the high cost and time required for granting FDA approval as the repurposed drug is generally an FDA approved drug, thus cutting the cost of the treatment [170]. The synergistic agents involved in modern combination therapies should ideally have a different pharmacological mechanism of action, exhibit no cross-resistance or overlapping toxicities and target tumor heterogeneity [171]. By integrating the knowledge and progress made in the field of mechanisms responsible for tumorigenesis, tumor microenvironment, therapy response and cancer heterogeneity, an effective treatment can be designed [169].

**Figure 9:**
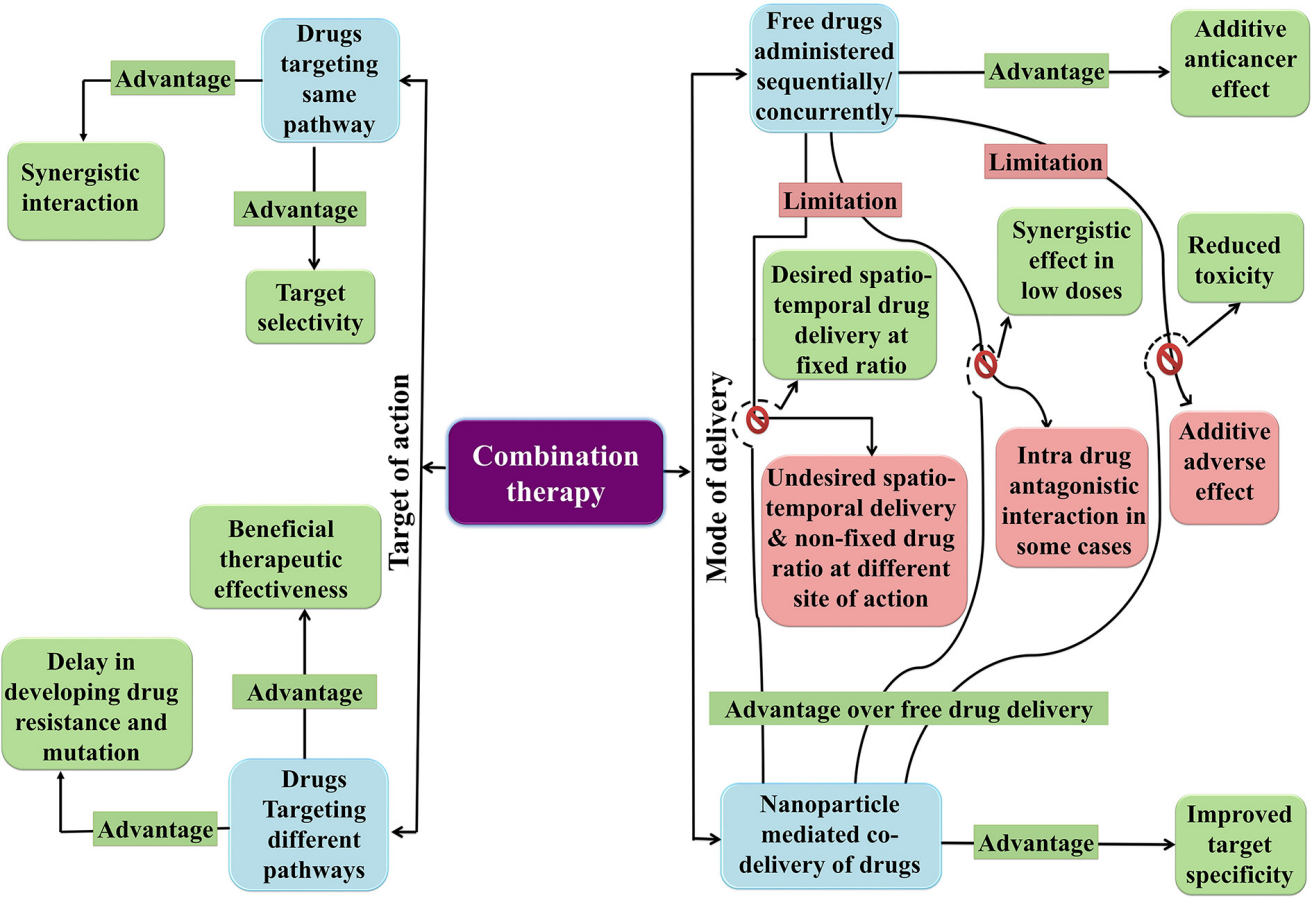
Rationale of nanoparticle-mediated combination therapy for cancer Combination therapy has been divided according to the target of action and mode of delivery. The figure represents the individual advantages of targeting same and different signaling pathways and the advantages of co-delivery over free drug delivery.

Nanoparticles have emerged itself as a potent tool to be used in combination therapy as they provide longer circulation time of biologically active drug, reduced toxicity, improved drug solubility, controlled release and has specific target potential [172]. Gurunathan and co-workers have elaborately described different types of nanoparticles mediated combination therapies [173]. The rationale for designing nanoparticle-mediated combination therapy for cancer treatment is provided in Figure 9. In Table 9, we have enlisted examples of nanoparticle-mediated combination therapy for treating different types of cancer and their advantages over monotherapy.

**Table 9:** Nanoparticle-mediated combination therapy with small molecules and nucleotide-based anticancer drugs

Drug 1	Mode of action of drug 1	Drug 2	Mode of action of drug 2	Indication	Comments on combination	Ref.
Sterically stabilized liposomal DOX	Cytotoxic	Liposome containing Bcl-2 antisense oligodeoxynucleotide, G3139	Gene silencing	*In vivo* melanoma model	Combination showed delayed tumor growth and increased accumulation of DOX in tumor site than monotherapy	[174]
Liposomal daunorubicin	Antitumor antibiotic	Cytarabine	Antineoplastic anti-metabolite	Patients having refractory or recurring acute myeloid leukemia	The combination has significant antileukemia activity with low toxicity. Liposomal encapsulation of daunorubicin changes the pharmacology profile to decrease toxicity and increase delivery to tumor sites	[175]
Liposome-entrapped, ends-modified raf antisense oligonucleotide (LErafAON)	Gene silencing	Cisplatin/epirubicin/mitoxantrone/docetaxel/gemcitabine	Chemotherapeutic agents	*In vivo* pancreas or pancreatic cancer model	Increased tumor growth inhibition as compared with single agents	[176]
aGD2-SIL(DOX) Sterically stabilized immunoliposomes(SIL) encapsulated with DOX, targeted to the disialoganglioside receptor GD2	Cytotoxic	NGR-SL(DOX) Sterically stabilized immunoliposomes (SIL) encapsulated with DOX, targeted to angiogenic endothelial cell marker aminopeptidase N by peptide NGR	Cytotoxic	*In vivo* neuroblastoma model	Considerable reduction of the angiogenic potential of various neuroblastoma xenografts	[177]
Non-pegylated liposomal Doxorubicin	Cytotoxic	Cyclophosphamide/ docetaxel	Cytotoxic	Patients with metastatic breast cancer	Use of non-pegylated liposomal Doxorubicin in combination with other drugs can be used for the first-line therapy against metastatic breast cancer	[178]
RGD SSL-DOX (RGD-SSL- RGD-modified sterically stabilized liposomes)	Cytotoxic	RGD-lipo-siRNA silencing P-glycoprotein	Gene silencing	*In vivo* mouse model of drug-resistant MCF7/A breast cancer tumor	Combination exhibited higher *in vitro* toxicity and greater inhibition of tumor growth	[179]
VEGF siRNA in polycation liposome-encapsulated calcium phosphate nanoparticles	Gene silencing	Doxorubicin	Cytotoxic	*In vivo* mouse model of breast cancer	Combination showed significant tumor growth and angiogenesis inhibition	[180]
Polymeric nanoparticle- encapsulated curcumin	Cytotoxic	Gemcitabine	Cytotoxic	*In vivo* mouse model of human pancreatic cancer	Enhanced tumor growth inhibition compared to single agents.	[181]
VOR-POEOMA Vorinostat encapsulated into poly(ethylene glycol) monomethacrylate (POEOMA)-based disulfide cross-linked nanogels.	Histone deacetylase inhibitor	ETOP-POEOMA Etopside encapsulated into poly(ethylene glycol) monomethacrylate (POEOMA)-based disulfide cross-linked nanogels.	Topoisomerase II inhibitor	Human cervical HeLa cancer cells	Combination showed enhanced synergistic cell killing efficiency	[182]
C60 fullerene	Cytotoxic	Doxorubicin	Cytotoxic	*In vivo* mouse model of lung cancer	Combination resulted in increased apoptosis in tumor cells and tumor growth inhibition	[183]
Anti Bcl-2 siRNA loaded polyethylenimine (PEI)-conjugated graphene oxide (PEI-GO)	Gene silencing	DOX loaded polyethylenimine (PEI)-conjugated graphene oxide (PEI-GO)	Cytotoxic	Human cervical HeLa cancer cells	Sequential delivery exhibited synergistic effect. Codelivery showed no significant synergistic effect on killing cancer cells	[184]
Palladium nanoparticles (PdNPs)	Cytotoxic	Trichostatin A (TSA)	Histone deacetylase inhibitor	Human cervical HeLa cancer cells	Combination exhibited synergistic interaction and also had an increased effect on cytotoxicity, oxidative stress and caspase-3/9 activity	[185]
Palladium nanoparticles (PdNPs)	Cytotoxic	Tubastatin A (TUB-A)	Histone deacetylase inhibitor	TNBC cell line MDA-MB-231	Combination had a more pronounced effect on the inhibition of HDAC activity and enhanced apoptosis of cells	[186]

In recent years, there has been an increase in the number of reports of multifunctional nanoparticle mediated combination therapy aided with peptides. Some of the advantages of peptide-mediated combination nanotherapeutics are tumor targeted delivery, presentation of tumor antigens for elicited immune response, sensitization of drug resistant cells and reduced side effects [187]. Mehrotra et al. and Mallick et al. have elaborately enlisted different types of peptide-based combination nanotherapeutics [187,188]. We have enlisted some of them in Table 10.

**Table 10:** Nanoparticle-mediated combination therapy having peptide-based anticancer drug

Peptide + Combination drug	Drug details	Indications	Comments	Ref.
dPPA peptide + paclitaxel prodrug + pheophorbide A	dPPA-1 peptide –NYSKPTDRQYHF (anti-PD-L1) paclitaxel- cytotoxic pheophorbide A- photosensitier	*In vivo*, breast cancer and lung metastasis	Increased NK cell and T-cell activation causing inhibition of complete lung metastasis and at least 10% decrease in primary tumor volume as compared with either alone or combination of paclitaxel and pheophorbideA	[189]
NuBCP-9 (Bcl-2 inhibitor) peptide + paclitaxel	NuBCP-9 -Ac-FSRSLHSLLGC-NH_2_ paclitaxel-cytotoxic	*In vivo*, breast cancer	Combination leads to reduced IC_50_ value (100-fold) in paclitaxel resistant cells and shows complete tumor inhibition in syngeneic mice model as compared with only paclitaxel	[190,191]
Acetylated rapeseed protein isolate derived peptides + DOX	From hydrolyzed ARPI peptides 3 bioactive peptides were screened. Sequences are AGS, PAS and YT. DOX- cytotoxic	*In vivo*, breast cancer	Enhanced cellular uptake and nuclear transport in comparison with free DOX. Increase in tumor inhibition and diminished DOX-associated cardiotoxicity.	[192]
PMI + BIM (Bcl-2 inhibitor) peptide + iNGR	PMI- p53 activating) peptide- TSFAEYWNLLSP BIM- Bcl-2 inhibitor) peptide-MRPEIWIAQELRRIGDEFNAYYARRV iNGR- CD-13) targeting peptide cyclic CRNGRGPDC	*In vivo*, colorectal cancer	Increased tumor inhibition (15%) with significant improvement in biosafety and reduced body weight loss compared with only DOX.	[193,194]
aFLT1 peptide + DOX	aFLT1 peptide- binds VEGFR1 isoform- GNQWFI-NH_2_ DOX-cytotoxic	*In vivo*, breast cancer	Two-fold increase in tumor inhibition	[195]
KLA + DOX	KLA - mitochondrial membrane disruptor) peptide KLAKLAKKLAKLAK DOX-cytotoxic	*In vivo*, colorectal cancer	Marked increase in tumor inhibition and mean survival time as compared with only DOX	[196]
R8 modified AVPI peptide with p53DNA+DOX	R8 modified AVPI peptide- cell penetrating apoptotic peptide- AVPIR_8_ p53 DNA- induces apoptosis DOX-cytotoxic	*In vivo*, resistant breast cancer model	4.4- and 2-fold increase in tumor inhibition in drug resistant mouse model as compared with equal and high free DOX dose, respectively	[197]
KLA peptide + chlorin e6	KLA peptide- membrane lysis peptide-D-(KLAKLAK)_2_ chlorin e6- generates singlet oxygen causing membrane disruption	*In vitro*, cervical carcinoma	Tenfold reduced IC_50_ value compared to only peptide	[198]
Wilms tumor gene (WT1) peptide-based vaccine+gemcitabine	WT1 peptide- target antigens for cancer immunotherapy CYTWNQMNL Gemcitabine- cytotoxic	Patients with advanced pancreatic cancer	Combination was found to be more effective than gemcitabine alone and combination therapy was well tolerated	[188,199]
Cep55/c10orf3_193+ Cep55/c10orf3_402 + Cep55/c10orf3_283	VYVKGLLAKI + EFAITEPLVTF + LYSQRRADVQHL antigenic peptides	Patients with colorectal carcinoma	Vaccination involving peptide mixture could be more efficacious compared with single peptide to treat colorectal carcinoma patients	[188,200]
“Peptide cocktail”	RNF43–721: NSQPVWLCL TOMM34–299: KLRQEVKQNL KOC1(IMP-3)-508 (KTVNELQNL) 3 peptides derived from oncoantigens VEGFR1–1084: SYGVLLWEI VEGFR2–169: RFVPDGNRI 2 peptides derived from angiogenesis factors	Patients with advanced colorectal cancer	Treatment with multiple peptides was well tolerated without systemic adverse effects. The median overall survival time was 13.5 months	[188,201]
7-peptide cocktail vaccine	RNF43: NSQPVWLCL TOMM34: KLRQEVKQNL FOXM1: IYTWIEDHF MELK: EYCPGGNLF HJURP: KWLISPVKI 5 tumor antigen-derived peptides VEGFR1: SYGVLLWEI VEGFR2: RFVPDGNRI 2 vascular endothelial growth factor receptor-derived peptides	Patients with metastatic colorectal cancer	Patients exhibiting positive cytotoxic T lymphocyte responses to all seven peptides had longer overall survival compared with other patients and this therapy is recommended for further trials	[188,202]
KIF20A-derived peptide + gemcitabine	KIF20A-peptide for trafficking of molecules and organelles during the growth of pancreatic cancer- KVYLRVRPLL Gemcitabine- cytotoxic	Patients with advanced pancreatic cancer	The disease control rate was 44%. The median survival time after first vaccination was 173 days and 1-year survival rate was 11.1%. No severe adverse effects of grade 3 or higher were observed	[188,203]
GV1001 + gemcitabine	GV1001 - telomerase peptide- EARPALLTSRLRFIPK Gemcitabine- cytotoxic	Patients with advanced pancreatic cancer	This combination appears to be safer with transient and weak immune responses	[188,204]
E75 + GM-CSF	E75- immunogenic peptide derived from the HER2 protein. KIFGSLAFL GM-CSF = granulocyte-macrophage colony-stimulating factor	Patients with node-positive or high-risk node-negative breast cancer	Therapy considered safe with a suggestion of clinical benefit. Has been licensed for commercial development	[188,205]
FNIII14+ Ara C	FNIII14-peptide derived from fibronectin- TEATITGLEPGTEYTIYVIAL Ara C – anti metabolic agent cytarabine/arabinosylcytosine	*In vivo* minimum residual disease (MRD) mice model	In mouse with MRD in bone marrow, 100% survival was achieved with this combination, whereas Ara C alone prolonged survival only slightly	[188,206,207]
D-K_6_L _9_ + IL-12	D-K_6_L_9_ - induces necrosis in cancer cells-Ac[D(K_6_L_9_)]-NH_2_ IL-12- pro-inflammatory cytokine-interleukin 12	*In vivo* murine melanoma model	This combination showed long-term tumor growth inhibitory effect	[188,208]
VEGFR2–169 + S-1 + cisplatin	VEGFR2–169-RFVPDGNRI S-1 - combination drug tegafur/gimeracil/oteracil cisplatin - cytotoxic	Patients with advanced gastric cancer	The combination therapy was highly effective and well tolerated in advanced or recurrent gastric cancer	[188,209]
LD8 + DOX	LD8 – gramicidin A inspired peptide Boc-^L^A-^D^V-^L^L-^D^A-^L^V-^D^A-^L^L-^D^W- OMe DOX- cytotoxic	*In vitro* TNBC cell line MDA-MB-231	LD8-DOX-NP induces G2 phase cell cycle arrest and apoptosis of MDA-MB-231	[210]

**Figure 10:**
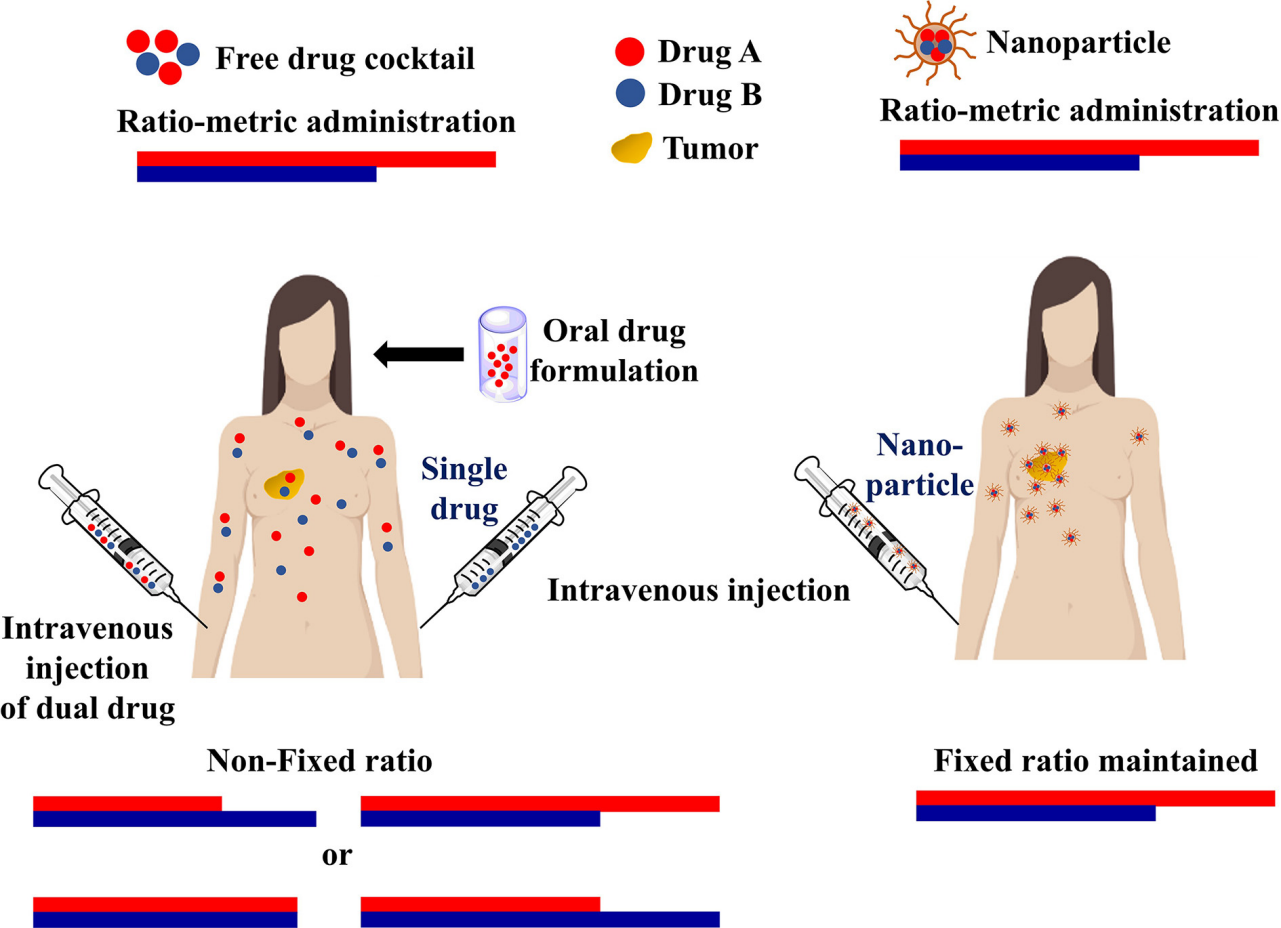
Schematic representation of nanoparticle-mediated ratiometric delivery of drug combination The scheme represents the pharmacokinetics and biodistribution of ratiometric drug combination. Drug combination delivered sequentially or concurrently either by similar or different route of administration show a non-fixed ratio in biodistribution of both the drugs. Nanoparticle mediated delivery of dual drugs maintain a fixed ratio of biodistribution of drug combinations resulting in higher therapeutic efficacy (This image was drawn based on the information provided in Zhang et al. 2016 [258], Figure 4). Adapted from “Body (female, teen)”, by BioRender.com (2021). Retrieved from https://app.biorender.com/illustrations/61d7dc25883c8d00a22cf5c8.

However, the current combination therapy treatment in cancer, especially in the case of metastatic breast cancer, is still loaded with flaws having moderate efficacy but additive toxicity [18]. Drugs, when administered separately without any modification, tend to not only cause an additive anticancer effect but also result in augmented adverse effects of each drug as well. For achieving synergistic interactions, a definite ratio of the two free drugs needs to be maintained. This ratio is generally determined in *in vitro* studies, and it is crucial that this ratio is to be maintained at tumor site. The problem arises because in *in vivo* model it becomes virtually impossible to deliver the determined ratio of free drugs in the tumor site. This is mainly due to the different pharmacokinetic properties, elimination and metabolism rates of individual drugs. Delivery of non-fixed ratio of drugs can give rise to antagonistic interactions and drug resistance in cancer cells [257]. For example, irinotecan and cisplatin (both cytotoxic drugs) show synergistic interaction at 4:1 ratio but strong antagonistic interaction at 1:1 ratio and in the case of many anticancer drugs, when the effective dose is below its optimum dose, it may give rise to drug resistant cells in tumor [257]. Another complication arises when the free drugs need to be administered by different routes and at different schedules. HER2 targeted combination therapy with Trastuzumab (TRZ, a monoclonal antibody binding to HER2 receptor) and lapatinib (a tyrosine kinase inhibitor blocking HER2 and EGFR pathways) have two different routes of administration. Lapatinib is administered daily as an oral formulation, while TRZ is given weekly as an intravenous drug. This difference in schedules and ways of administration of these two drugs makes the management of pharmacokinetic and pharmacodynamic profiles more challenging and virtually impossible to achieve uniform temporal and spatial co-delivery [18]. Most standard chemotherapy guidelines prefer sequential delivery of free drugs over concurrent delivery due to toxicity issues. This also prevents the right temporal delivery of drugs with dissimilar pharmacokinetic properties [258]. To address these issues, nanoparticle-mediated co-delivery of those drugs may ensure the desired spatio-temporal delivery and controlled release of drugs while maintaining the synergistic fixed ratio (Figure 10) [258]. Nanocarrier mediated co-encapsulated drugs having physically different properties show similar pharmacokinetic profiles, extended drug half-life, solubility, tumor accumulation and synergistic drug interactions when compared with free drugs administered sequentially or concurrently [18,257]. Cytrabine (an antimetabolite chemotherapeutic drug) and danorubicin (a cytotoxic chemotherapeutic drug) has distinct pharmacokinetic properties as free drugs but when co-encapsulated in liposomal nanocarrier as Vyxeos® exhibit similar pharmacokinetic profile [257]. Nanocarrier mediated co-delivery of drugs enables targeting multiple signaling pathways, overcoming drug resistance and immunosuppression by cancer [257].

Various delivery systems widely used for co-delivery of two drugs include liposomes, dendrimers, polymeric nanoparticles and water-soluble polymer conjugates. Lee et al. and Gurunathan et al. have elaborately reviewed nanoparticle-mediated co-delivery of two or more drugs for cancer therapy as described in Table 11 [18,173].

**Table 11:** Nanoparticle mediated co-delivery of drugs for cancer therapy

Carrier composition	Therapeutics	Indication	Status	Targeting	Ref.
**Liposome based co-delivery of drugs against cancer**					
PEG-Liposome	Topotecan + Vincristine	Brain cancer	*In vivo*	Passive	[18,211]
Polymer-caged nanobins (PCN); liposome surrounded by cholesterol-terminated poly(acrylic acid)	Cisplatin + Doxorubicin	Various cancers	*In vitro*	Passive	[18]
Liposome	Cytarabine + Daunorubicin	Acute myeloid leukemia	Phase II	Passive	[18,212]
Liposome	Irinotecan + Floxuridine	Colorectal cancer	Phase II	Passive	[18,213,214]
The mixture of two Liposomes	Irinotecan + Cisplatin	Small-cell lung cancer	*In vivo*	Passive	[18,214]
PEG-Liposome	Quercetin + Vincristine	Hormone- and TRZ insensitive breast cancer	*In vivo*	Passive	[18,215]
Cationic, anionic PEG Liposome	VEGF/ c-myc siRNA + Doxorubicin	MDR-breast cancer	*In vivo*	Passive	[18,216]
Liposome	6-Mercaptopurine + Daunorubicin	Acute myeloid leukemia	*In vitro*	Passive	[18,217]
**Dendrimer based co-delivery of drugs against cancer**					
G5 PAMAM dendrimer (G5-Generation 5, PAMAM-poly(amidoamine))	Antisense-miRNA21 +5-fluorouracil	Glioblastoma	*In vitro*	Active; miRNA overexpression	[18,218]
Aptamer-G4 PAMAM dendrimer conjugates (G4-Generation 4)	Unmethylated CpG-oligonucleotides+ Doxorubicin	Prostate cancer	*In vivo*	Active; a single-strand DNA-A9 prostate-specific membrane antigen, RNA aptamer hybrid	[18,219]
Dendritic PEG H_2_N–PEG–dendrimer– (COOH)_4_	Paclitaxel + alendronate	Cancer bone metastasis	*In vivo*	Both passive and active Active by alendronate molecule	[18,220]
RGDfK-G3 Poly-lysine dendrimer (G3-Generation 3)	Doxorubicin + siRNA	Glioblastoma	*In vitro*	Active; α_v_β_3_ integrin	[18,221]
Folate-G5 poly -propyleneimine dendrimer with ethylenediamine core (G5-Generation 5)	Methotrexate + all-trans-retinoic acid	Leukemia	*In vitro*	Active; folate receptor	[18,222]
**Polymer based co-delivery of drugs against cancer**					
PEG-PLGA	Lonidamine + Paclitaxel	Multiple drug resistant (MDR) breast cancer	*In vitro*	Active; EGFR	[18,223]
Methoxy PEG-PLGA	Doxorubicin+ paclitaxel	Various cancers	*In vitro*	Passive	[18,223]
PEG-PLA (PLA-poly(d,l, lactic acid))	Paclitaxel, Etoposide, or Docetaxel + 17-AAG	Various cancers	*In vitro*	Active; HSP90	[18,224]
PEG-PLA	Combretastatin A4 + Doxorubicin	Various cancers	*In vitro*	Active; angiogenesis	[18,225]
PDMAEMA-PCL- PDMAEMA poly(N,N-dimethylamino-2- ethyl methacrylate)- polycaprolactone-poly (N,N-dimethylamino- 2-ethyl methacrylate)	Paclitaxel + VEGF siRNA	Prostate cancer	*In vitro*	Active; VEGF	[18,226]
PEG-DSPE/PLGA	Combretastatin + Doxorubicin	Lung carcinoma	*In vitro*	Passive	[18,115]
PEG-PLA and PEG-DSPE/TPGS (TPGS- tocopheryl polyethylene glycol)	Paclitaxel + 17-AAG(HSP90 inhibitor)	Ovarian cancer	*In vitro*	Active; HSP90	[18,227]
P(MDS-co-CES) poly (N-methyldietheneamine sebacate)- co-[(cholesteryl oxocarbonylamido ethyl) methyl bis(ethylene) ammonium bromide]	Paclitaxel + Interleukin-12 or Bcl-2 siRNA	Breast cancer	*In vivo*	Active; Bcl-2	[18,228]
PEG-b-PHSA PEG-block-poly(N-hexyl stearate l-aspartamide)	Doxorubicin + Wortmannin	Breast cancer	*In vitro*	Passive	[18,229]
PLN formulation ((DG)n) (Polymer lipid hybrid nanoparticles (NP) co-loaded with DOX and GG918)	Doxorubicin + GG918	Breast cancer	*In vitro*	Passive	[230]
PLGA	Vincristine + Verapamil	Hepatocellular carcinoma	*In vitro*	Passive	[18,231]
PLGA	Paclitaxel + Tariquidar	Breast cancer	*In vivo*	Active; functionalized with biotin	[173,232]
PLGA	Rapamycin + piperine	Breast cancer	*In vitro*	Passive	[173,233]
PACA polyalkylcyanoacrylate	Doxorubicin + Cyclosporine A	Various cancers	*In vitro*	Passive	[18,234]
PEG outer shell, middle PCL and inner CPCL core (PCL- polycaprolactone; CPCL- carboxylic functionalized PCL)	Doxorubicin +Cisplatin	Breast cancer	*In vitro*	Passive	[173,235]
Bradykinin-potentiating peptide decorated chitosan nanoparticle	Bradykinin-potentiating peptide + bioreductively sensitive platinum (IV) compound which becomes cisplatin in intracellular reductive environment	Hepatocellular carcinoma	*In vivo*	Passive	[188,236]
**Polymeric micelles based co-delivery of drugs against cancer**					
MPEG-b-P(LA-co-MCC) (MPEG-b-P(LA-co-MCC)) - methoxy poly(ethylene glycol)-block-poly(1-lactide -co-2-methyl-2- carboxyl-propylene carbonate)	Paclitaxel +Cisplatin	Cervical cancer	*In vivo*	Passive	[173,237]
PEG–PLL–PLLeu poly(ethylene glycol)- *b*-poly(L-lysine)-*b*- poly(L-leucine)	Docetaxel + Bcl-2 siRNA	Breast cancer	*In vivo*	Passive	[173,238]
PCL-b-P(OEGMA-co- AzPMA POEGMA- poly(OEGMA); OEGMA- oligo(ethylene glycol) ethyl methacrylate; co- copolymer; AzPMA- 3-azidopropyl methacrylate	Doxorubicin+ platinum(IV)	Cervical cancer and melanoma	*In vitro*	Passive	[173,239]
DA3 (deoxycholic acid-conjugated PEI)	Paclitaxel + XIAP siRNA	Colorectal cancer	*In vivo*	Passive	[173,240]
Self-assembled polymeric micelles	Paclitaxel + survivin siRNA	Ovarian cancer	*In vivo*	Passive	[173,241]
P–H/M (methoxy poly(ethylene glycol)– poly(caprolactone) micelles)	Paclitaxel + Honokiol	Breast cancer	*In vivo*	Passive	[173,242]
Crosslinked PEG-*b*-pAsp-*b*-pTyr	Docetaxel +lonidamine	Breast cancer	*In vivo*	Passive	[173,243]
**Water-soluble polymer conjugate-based co-delivery of drugs against cancer**					
HPMA copolymer	Doxorubicin+ dexamethasone	General cancer	*In vivo*	Passive	[18,244]
HPMA copolymer	TNP-470 + Alendronate	Bone metastasis	*In vivo*	Active; bone	[18,245]
HPMA copolymer	Paclitaxel + Alendronate	Bone metastasis	*In vivo*	Active; bone	[18,118]
Branched PEG	Epirubicin + Nitric oxide	Colon cancer	*In vivo*	Passive	[18,246–248]
Branched PEG	Camptothecin + BH3 domain peptide	Ovarian primary tumor and metastatic malignant ascites	*In vivo*	Active; luteinizing hormone-releasing hormone	[18,249]
HPMA copolymer	Trastuzumab + PKI166	HER2 overexpressed breast cancer	*In vitro*	Active; HER2	[18,250]
HPMA copolymer	6.4 wt% gemcitabine + 5.7 wt% of Doxorubicin + 1.0 mol% tyrosinamide	Prostate cancer	*In vivo*	Passive	[173,251]
**Microsphere-based co-delivery of drugs against cancer**					
Double-walled microspheres,PLGA core surrounded by PLLA shell (PLLA- poly(L-lactic acid))	Doxorubicin+ Chitosan p53 DNA	Hepatocellular carcinoma	*In vitro*	Passive	[173,252]
**Carbon nanoparticle and carbon-based nanosystem based co-delivery of drugs against cancer**					
Nanodiamond	Paclitaxel + Cetuximab	Colorectal cancer	*In vivo*	Active, epidermal growth factor receptor positive cells	[173,253]
PEGylated lipid bilayer-wrapped nano-graphene oxide (GOLDR)	Doxorubicin + Rapamycin	Breast cancer	*In vitro*	Passive	[173,254]
**Metallic nanoparticle-based co-delivery of drugs against cancer**					
Silver nanoparticles (SN-AK-DOX) (SNs- silver nanoparticles; AK- sanazole)	Sanazole + Doxorubicin	Lymphoma	*In vivo*	Active, hypoxic cells	[173,255]
Gold nanoparticles	Doxorubicin+ Cisplatin, +Capecitabine	Hepatocellular carcinoma	*In vitro*	Passive	[173,256]

In some instances, co-delivery of drugs in a single nanoparticle suffers from drug leakage and poor loading efficacy [259]. To overcome this problem, researchers have conjugated two anticancer drugs via suitable covalent linkages and then subsequently encapsulated the conjugate in a nanoparticle. For example, Aryal et al. have used ester linkage for conjugating paclitaxel and gemcitabine and loaded the drug conjugate into a PLGA nanoparticle. Hydrolysis of this ester linkage at mildly acidic pH (pH 6) of endosomal environment resulted in two separate functional drug fragments [260]. Matlapudi et al. have conjugated Imatinib mesylate (abbreviated as IM, a tyrosine kinase inhibitor) and 5-fluorouracil (abbreviated as FU, an antimetabolite) by hydrolysable succinyl linker (abbreviated as Su) which forms amide linkage with each drug. This drug conjugate IM-Su-FU was incorporated in a human serum albumin (HSA) nanoparticle. This HSA encapsulated drug conjugate (IM-Su-FU) nanoparticle exhibited higher anticancer efficacy in *in vivo* lung cancer model compared to free drugs and only IM-Su-FU conjugate. Pharmacokinetic analysis of this nanoparticle exhibited improved elimination rate, half-life and mean residence time (MRT) than free drug and only IM-Su-FU conjugate [261].

## Challenges and future prospect of nanoparticle-mediated combination therapy

Nanoparticle-mediated combination therapy has shown great potential in treating metastatic and drug resistant cancer [262]. Combination therapies involving cytotoxic drugs, signal transduction inhibitors, immunotherapeutic drugs, epigenetic agents and priming with apoptotic drugs have shown promising possibilities for cancer therapeutics [258]. The success of nanoparticle mediated combination owes to its “3R” delivery principle, i.e., right place, right dose and right time. To gain the benefits of nanocarrier mediated combination therapy, different databases and drug development platforms are developed. EMBASE® and Ovid MEDLINE® are databases used for the co-delivery of drugs. The drug combination development platform CombiPlex screens dual drugs and makes nanoscale formulation for nanocarrier mediated co-delivery of drugs. CombiPlex platform first determines the synergistic ratio of free drugs and then chooses a suitable nanocarrier to coordinate the pharmacokinetics of the free drugs and ensures the drugs reach the tumor site in the desired ratiometric manner. CPX-351, developed from CombiPlex platform is an FDA approved drug used for treating AML in adults. CPX-351 comprises cytarabine and daunorubicin in a synergistic ratio encapsulated in a liposomal carrier [257].

Although nanoparticle-mediated combination therapy has immense potential, but the regime of nanoparticle-based combination therapy is far from being able to cure metastatic cancers. 0.7% is the median efficiency of delivery of the injected nanoparticle to the desired tumor site [24]. Additionally, the five-year survival rates of most malignant cancer are still quite low and most combination approaches still depend on cytotoxic approaches instead of molecularly targeted anticancer agents or nanoparticle mediated combination approach [263]. Saptura et al. has shown antagonistic drugs interactions used in combination can suppress the clonal expression of singly-resistant cells in *in silico* model [264]. So antagonistic drug interactions should also be investigated and not completely discarded. Although around five thousand clinical trials are ongoing worldwide for the development of new combination therapy, Palmer and Sorger, 2017 claims that most of the combination therapy used follows the independent action model and provide therapeutic benefit due to patient-to-patient variability rather than additive or synergistic drug interactions [265,266].

One of the key challenges in developing nanoparticle-based targeted therapy is the issue of toxicity of treatment combinations. Seemingly rationally developed and assumed to be safe combination therapy in preclinical model may fail in clinical trials due to toxicity, especially in the case of synergistic drugs which can lead to synergistic toxicity as well due to similar mechanism of action or due to auto immune response of healthy tissue [169]. Mathematical modeling has been helpful in predicting toxicity in pre-clinical trials and can be used as an efficient tool for predicting the toxicity of drug combinations in a patient-specific manner [169]. Toxicity of the drug nanocarrier also needs to be considered. Another hurdle in the success of combinatorial nanotherapeutics to be translated into clinics is the lack of patients who are interested in participating in clinical trials. Approximately 40% of cancer trials fail due to the scarcity of patients [169]. Cancer is a highly heterogeneous disease and cell type population in a particular type of malignant tumor varies from patient to patient. For clinical trials to have higher success rates, efficient biomarkers need to be identified first. Additionally, clinical trials should be performed on patients with a similar level of biomarker expression. But due to the lack of patients enrolled in a clinical trial, such arrangements are not possible. Due to the insufficiency of suitable patients for performing clinical trials, precise preclinical models need to be developed. Researchers need to look beyond immune-competent mice model and use patient-derived xenograft *in vivo* models, humanized mouse models and patient-derived organoids for a greater chance of success in clinical trials and such models can also be used as an efficient tool for predicting the toxicity of drug combinations in a patient specific manner [169].

## Nanomedicine and combination nanomedicine for cancer immunotherapy

Immunotherapy is a monumental breakthrough recently included in the existing therapeutic armamentarium against cancer. It utilizes body’s own immune system to fight cancer. William Coley, known as the father of immunotherapy, first attempted to treat cancer utilizing the immune system. However, this field got attention when James P. Allison and Tasuku Honjo got the Nobel prize for cancer immunotherapy in the year 2018. Judy Perkins having stage IV metastatic breast cancer, was the first lady to be cured successfully by immunotherapy [267]. Current cancer immunotherapy comprises cytokine therapy, antibody-based therapy and adoptive cell therapy. However, complex tumor microenvironment limits the efficacy of immunotherapy. In the tumor microenvironment, tumor cells can polarize tumor associated macrophages (TAM) toward pro-tumorigenic M2 macrophages while decreasing antitumorigenic M1 macrophages to facilitate tumor progression. Moreover, cancer cells help to activate immune checkpoints leaving the T cells in a state of anergy (lack of responsiveness to an antigen). Cancer immunotherapy inhibits immune checkpoints to overcome the T-cell anergy, thereby its activation against cancer. Ipilimumab inhibits the cytotoxic T-lymphocyte–associated antigen 4 (CTLA-4) checkpoint in T cells leading to its activation. Similarly, programmed cell death protein 1 (PD-1) in T cells is inhibited by FDA-approved anti PD-1 antibody pembrolizumab and nivolumab. Cancer cells overexpress the immune checkpoint protein programmed death-ligand 1 (PD-L1). The PD-L1 inhibiting antibodies atezolizumab, avelumab, durvalumab are also approved by FDA [268]. These antibodies block the inhibitory signal induced by interaction of PD-1 of T cell to PD-L1 of the cancer cell. Multiple colony stimulating factor receptor 1 (CSF-1R) inhibitors are currently in clinical trials for their ability to polarize tumor associated macrophages to anti-tumorigenic M1 phenotype [13].

Peptide-based drugs/vaccines can hugely advance cancer immunotherapy. Peptides can be designed in a more rationalized manner to target desired molecule of tumor or the tumor microenvironment. Yin et al. have designed a peptide (IQIREYKRCGQDEERVRRECKERGERQNCHYVIHKEGNCYVCGIICL) mimicking the native structure of the PD-1 molecule, which inhibited the interaction between PD-L1 of cancer cells and PD-1 of immune cells. This peptide was developed as a potent drug for cancer immunotherapy [269]. Hazama et al. also developed a peptide-based macrocyclic peptide (c[Ac-^D^YRYSAVYSIHPSWC]G) inhibiting the interaction between cluster of differentiation 47 (CD47) of cancer cell and signal regulatory protein α (SIRPα) of macrophages and showed its *in vivo* efficacy [270]. Peptide-based cancer vaccines can specifically stimulate cancer specific T cell response [271]. Peptide-based (9 amino acid residues) vaccine GP2 (IISAVVGIL) is currently in phase 2 clinical trial against breast cancer [272]. Several other examples of peptide vaccines combined with other drugs are highlighted in Table 10.

**Figure 11:**
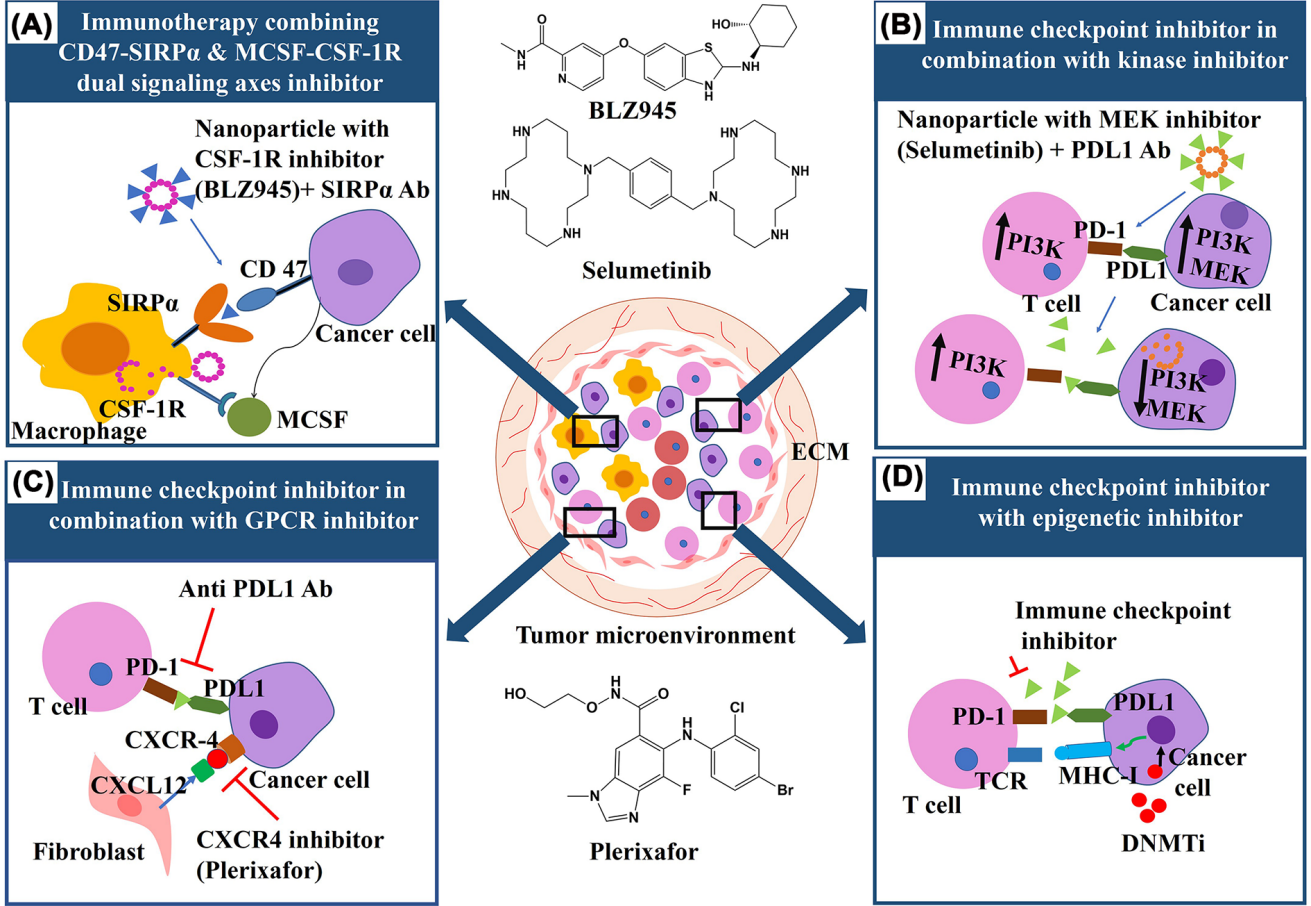
Next-generation combination nanomedicine for immunotherapy (**A**) CD47 (cluster of differentiation 47; a transmembrane protein) is overexpressed on cancer cells and binds to SIRPα (signal regulatory protein α; a regulatory membrane glycoprotein) on immune cells to inactivate immune cells and escape immune surveillance. Similarly, MCSF (macrophage colony stimulating factor; a secretory protein) released from cancer cells which binds to CSF-1R (colony stimulating factor 1 receptor; a transmembrane protein) on immune cells and inactivates immune cells. A supramolecular nanoparticle, comprising anti-SIRPα antibody with a small molecule inhibitor (BLZ-945) of CSF-1R inhibits both the signaling axes simultaneously and shows anti-tumor immune response. (**B**) Nanoparticle having immune checkpoint inhibitor and a small molecule-based kinase inhibitor causes targeted disruption of kinase signaling only in cancer cells while keeping the PI3K and MAPK pathways of immune cells untouched. This strategy enables inhibition of kinase signaling. (**C**) Chemokine CXCL12 (C-X-C motif chemokine ligand 12), secreted from fibroblast cells binds to CXCR4 (C-X-C chemokine receptor 4), a G-protein-coupled receptor from cancer cells and promotes immunosuppressive tumor microenvironment. Combination immunotherapy with inhibitors of CXCR4/CXCL12 axis can amplify the antitumor efficacy. (**D**) combination of epigenetic inhibitor (Zebularine, a DNMTi) with immune checkpoint inhibitor of PD-L1 can potentiate combination immunotherapy.

Researchers have reported a number of combinatorial approaches where immunotherapeutic drugs are combined with the conventional cancer therapeutics such as chemotherapy, RNAi therapy, photothermal, photodynamic and radiotherapy as described in Tables 12-14 and Figure 11.

**Table 12:** Combination nanomedicine having immunotherapeutic drugs with chemotherapeutic drugs

Chemotherapeutic drugs	Immunotherapeutic drugs	Nanoparticle delivery system	Cancer Type	Outcome	Ref.
Doxorubicin	Anti–PD-1 antibody	Synthetic high-density lipoprotein (sHDL) like nanodiscs loaded with DOX	CT26 and MC38 mouse colon carcinoma	Induced strong anticancer immunity and sensitized tumors to immune checkpoint blockade	[273]
Doxorubicin	Cytosine–phosphate–guanosine oligonucleotides (CpG-ONT) (Immune-stimulating agent)	An RNA aptamer (recognizing a prostate-specific membrane antigen (PSMA)) bioconjugated with a dendrimer attached with CpG-ONTs loaded with DOX	*In vivo* and *in vitro* models of prostate cancer (22RV1)	Showed excellent antitumor efficacy, immune stimulation and target specificity	[219]
Paclitaxel	Toll-like receptor-7 (TLR-7) agonist-imiquimod	Poly (γ-glutamic acid) (γ-PGA) micro-dispersion system of drugs	*In vitro* and *in vivo* mouse melanoma (B16-F10)	Showed robust immunogenic tumor cell death followed by inhibition of secondary tumors also	[274]
Paclitaxel	TLR-4 agonist bacterial endotoxin Lipopolysaccharide (LPS)	Co-encapsulation by PLGA-based nanoparticle	*In vitro* and *in vivo* murine melanoma model (B16-F10)	Tumor volume was found 40% less and immune activation was observed	[275]
Paclitaxel	Cytosine–phosphate–guanosine oligodeoxynucleotides (CpG ODNs) and IL-10 siRNA (Immune-stimulating agents)	PLGA-based nanoparticles	Murine melanoma model (B16-F10)	Efficiently inhibited tumor growth and increased the animal survival rate	[276]
Cisplatin	Cytosine–phosphate–guanosine (CpG) (Immune-stimulating agent)	Liposome	Murine melanoma model (B16-F10)	Strong synergistic effect which increased apoptosis and reduced tumor growth	[277]
Mitoxantrone treated CT26 cancer cells decorated with Cytosine– phosphate–guanosine (CpG) (a potent TLR9 agonist) loaded nanoparticle	Anti-PD-1 antibody	Hyaluronic acid-cationic lipid nanoparticle was loaded with CpG	Murine model of melanoma and colon carcinoma (B16-F10-OVA and CT26)	Complete tumor regression in almost 78% of CT26 tumor-bearing mice and long-term immunity against tumor recurrence	[278]
Doxorubicin and immune stimulating agent CpG-loaded microparticles	Immune checkpoint inhibitor antibodies anti-CTLA-4 and anti-OX40	PLGA-based microparticles	Mouse lymphoma (EL4, E20) and mouse melanoma (B16-fLUC) models	Generated systemic immune responses that suppressed injected and distant tumors in a murine B lymphoma model, leading to tumor-free mice Reduced tumor burdens	[279]
Doxorubicin	Immunotherapeutic agent interferon-γ (IFN-γ)	PLGA-based thermosensitive nanoparticle	Murine melanoma model (B16-F10)	Prolonged circulation time, sustained drug release, excellent synergistic antitumor efficiency against B16F10 tumor bearing mice	[280]
Paclitaxel	Interleukin-2	Hydroxypropyl-β- cyclodextrin acrylate and two opposite charged chitosan derivatives based nanogels coated by RBC membrane	Murine melanoma model (B16-F10)	Enhanced antitumor activity with improved drug penetration and increased antitumor immunity	[281]
Doxorubicin (DOX), all-trans retinoic acid (ATRA),	Interleukin-2	Lipid-coated biodegradable hollow mesoporous silica nanoparticle (dHMLB)	Murine melanoma model (B16-F10)	Significant tumor growth and metastasis inhibition	[282]

**Table 13:** Combination nanomedicine having immunotherapeutic drugs with siRNA

Gene therapy	Immunotherapeutic drugs	Nanoparticle delivery system	Cancer	Outcome	Ref.
Signal transducer and activator of transcription-3 (STAT3) silencing siRNA	TLR-7 agonist imiquimod, R837 (immune response modifier)	PLGA NPs	Murine T-cell lymphoma model (EG7-OVA)	Inhibited tumor growth efficiently	[283]
IL10-silencing siRNA	CpG ODN	Cationic PLGA-PEI microparticles	Murine model of B cell lymphoma (A20)	Better immune protection of an idiotype DNA vaccine	[284]
TGF-β silencing siRNA	A mannose-modified lipid-calcium-phosphate Nanoparticle based vaccine containing tumor antigen (Trp 2 peptide) and adjuvant (CpG oligonucleotide)	Liposome-protamine- hyaluronic acid (LPH) NP	Murine melanoma model (B16F10)	Boosted the vaccine efficacy and inhibited tumor growth by 52%	[285]
IL-6 silencing siRNA	Radiofrequency thermal ablation	Micelle like nanoparticle	Mouse breast adenocarcinoma (R3230 and MATBIII)	Reduced tumor growth	[286]
PD-L1 silencing siRNA	Photodynamic therapy	Acid-activatable cationic micelle	Murine melanoma model (B16F10)	Significantly enhanced efficacy for inhibiting tumor growth and distant metastasis	[287]

**Table 14:** Combination nanomedicine of immunotherapeutic drugs with photothermal, photodynamic and radiotherapy

Photothermal/ photodynamic/ radiotherapy	Immunotherapeutic drugs	Nanoparticle delivery system	Cancer	Outcome	Ref.
**Combination of immunotherapeutic drugs with photothermal therapy**					
Photothermal ablation (near infrared light)	Immunoadjuvants oligodeoxynucleotides containing the cytosineguanine (CpG) motifs	Chitosan coated hollow Copper Sulfide nanoparticles	Murine breast cancer model (EMT6-OVA, EMT6)	Combined photothermal immunotherapy is more effective than immunotherapy/ photothermal therapy alone in mouse breast cancer model	[288]
Photothermal ablation (near infrared laser)	Adoptive T cell therapy	Gold nanoshell	Murine melanoma (B16-F10)	Prevents primary tumor recurrence post-ablation, inhibited tumor growth at distant sites, and abrogated the outgrowth of lung metastases	[289]
Photothermal ablation	Anti-CTLA-4 antibody	Single-walled carbon nanotube	Murine model of breast cancer (4T1)	Tumor metastasis prevented	[290]
Photothermal ablation	Gold nanostar	Anti-PDL1 antibody	Mouse bladder cancer (MB49)	Both primary and distant tumors were safely eradicated	[291]
**Combination immunotherapeutic drugs with photodynamic therapy**					
Photosensitizer pyropheophorbide-lipid conjugate (pyrolipid) in the shell and oxaliplatin in the core	Anti PD-L1 antibody	Nanoscale coordination polymer (NCP) core-shell nanoparticles	Murine colorectal tumor (CT26 and MC38)	This combination causes regression of both primary and distant tumors via induction of strong cancer specific immune response	[292]
Photosensitizer pyrolipid (ZnP@pyro)	Anti PD-L1 antibody	Zn-pyrophosphate (ZnP) nanoparticles	Murine breast cancer model (4T1)	Complete eradication of primary and distant tumors via systemic cancer specific cytotoxic T-cell response	[293]
Chlorin e6 (Ce6), a photosensitizer and imiquimod (R837), a Toll-like-receptor-7 agonist	Anti-CTLA-4 antibody	Upconversion nanoparticles (UCNPs)	Murine colon carcinoma (CT26)	Eliminates NIR laser exposed tumors but causes strong anticancer immunity to inhibit distant tumors also	[294]
TBC-Hf (derived fromn tetra(pbenzoato)chlorin and Hf) enabled photodynamic therapy	Small-molecule inhibitor of indoleamine 2,3-dioxygenase (IDO)	Chlorin-based nanoscale metal−organic framework (nMOF)	Murine colorectal models (CT26 and MC38)	Effective local and distant tumor rejection in colorectal cancer models	[295]
**Combination immunotherapeutic drugs with radiotherapy**					
Radiation therapy	Cowpea-mosaic virus	Cowpea-mosaic virus nanoparticle	Murine ovarian cancer (ID8- Defb29/Vegf-A-Luc cells)	Resulted in improved tumor growth delay and an increase in tumor infiltrating lymphocytes (TILs)	[296]
Radiotherapy	Small molecule based indoleamine 2,3-dioxygenase (IDO) inhibitor	Hafnium (Hf)-based nanoparticle	Mouse models of breast and colorectal cancer	Eradication of local and distal tumors in *in vivo* models	[297]

## Chemotherapy with immunotherapy

Combination chemotherapy with cancer immunotherapy is a wide area of interest. In phase III clinical trial, albumin nanoparticle bound chemotherapeutic drug paclitaxel (Abraxane®) combined with atezolizumab (Tecentriq®; FDA approved antibody of PD-L1) has recently demonstrated its efficacy in patients with advanced triple-negative breast cancer (TNBC) [22,298]. However, some patients showed grade 3 (moderate to severe) or grade 4 (life threatening symptoms) immune related adverse events [298]. Hence, combination of nanoparticle-mediated chemo-immunotherapy may facilitate targeted and localized delivery to increase the therapeutic index, reduce off-target toxicity and incidences of immune related adverse events. Tables 12 shows some examples of preclinical studies on combination of nanoparticle mediated chemotherapy and immunotherapy.

## RNAi therapy with immunotherapy

RNA interference (RNAi) therapy with siRNA, microRNA (miRNA) and short hairpin RNA (shRNA) is utilized to silence genes of specific signaling molecules, cytokines and chemokines. However, this therapy possesses certain limitations like rapid degradation of RNA-based drugs in circulation due to presence of nucleases, renal clearance, poor cellular uptake because of anionic nature, etc [299]. Therefore, nanoparticles have been used to circumvent such barriers. Gene therapy has been utilized in targeting immune checkpoints like PD-1-PD-L1 pathway [300]. Also, these can be used with other cancer treatment modalities to induce immune stimulation against cancer. Few examples are given in Table 13 where combination nanomedicine having immunotherapeutic drugs showed enhanced efficacy.

## Photothermal, photodynamic and radiotherapy with immunotherapy

Photothermal, photodynamic and radiotherapies are also used in combination with immunotherapy. In photothermal therapy, cancer cells can be destroyed and eliminated from the tumor tissues at 40–44°C temperature due to DNA damage, protein denaturation and disruption of cellular membrane [20]. However, it requires high temperature for complete cell death, thus large tumors are prone to relapse. Thus combining photothermal therapy with immune-stimulating agents and nanoparticles can synergistically induce anti-tumor efficacy for the treatment of large established tumors and distant metastases.

Photodynamic therapy comprising combination of light with photosensitizers leads to the generation of reactive oxygen species (ROS) and subsequently damage of subcellular organelles [301]. However, as a monotherapy, it does not efficiently regress the tumor in an immunosuppressive tumor microenvironment [20]. It induces immunosuppression by the release of immunosuppressive cytokines. It provokes damage to the normal cells by releasing the self-antigens [302]. Thus, to balance the immunosuppressive effects of mono-photodynamic therapy, immunotherapy needs to be combined with it.

In radiotherapy, high energy ionizing radiation such as X-rays are utilized to cause free radical generation mediated DNA and cellular damage leading to cellular death [303]. However, radiation therapy helps to develop anticancer therapy, its solo use promotes immunosuppressive environment around tumor by recruiting immunosuppressive T_reg_ cells [304]. It causes increased production of immune-inhibitory molecules such as PD-L1 and transforming growth factor-β (TGFβ) [305]. Thus, to overcome these problems of radio-monotherapy, combination of radiotherapy with immunotherapy is implicated. Some examples are given in Table 14.

## Next-generation combination nanomedicine for immunotherapy

Immune checkpoint inhibitors were initially used in combination with other conventional chemotherapeutic drugs or radiotherapy (Tables 12-14) to potentiate the tumor regression. At present, nanomedicines for immunotherapy combines mechanistically inspired drug which can demonstrate a synergistic effect. Sengupta and co-workers have designed a supramolecular bifunctional nanomedicine comprising amphiphiles which inhibits interaction of CD47 of cancer cell and SIRPα of macrophages and simultaneously the interaction of macrophage colony stimulating factor (MCSF) and colony stimulating factor 1 receptor (CSF-1R). This nanomedicine increases the M2 to M1 repolarization within the tumor microenvironment and improves the anticancer and antimetastatic efficacy in melanoma and breast cancer model [306]. Mitragotri and co-workers designed a class of phagocytosis-resistant discoidal particles containing interferon-γ (IFN-γ). These particles showed efficient adherence to macrophages and directed their polarization toward anti-tumor M1 phenotype in murine breast cancer model [307]. Other such combinations are described in the next section.

## Combination immunotherapy with signaling pathways inhibitors

Next-generation cancer immunotherapy needs to be patient-specific to enhance its antitumor efficacy. Immunotherapeutic drugs can be combined with signaling pathway inhibitors for better synergistic outcomes and to amplify the efficacy of personalized medicine [308]. MEK inhibitors are used in combination with PD-L1 antibodies as MEK inhibition causes up-regulation of PD-L1 in cancer cells. In cancer, the dysregulated PI3K-AKT pathway also regulates the PD-L1 expression [309]. Kulkarni et al. designed a nanomedicine combining PD1-PDL1 immune checkpoint inhibitor with kinase (MEK and PI3K) inhibitors for enhanced anti-tumor efficacy [81]. It has also been reported that second generation anti-histaminic drugs like cetirizine, etc., in combination with immunotherapy, have improved the anticancer efficacy [310].

## Combination immunotherapy with GPCR inhibitor

Combination of G-protein-coupled receptor (GPCR) inhibitors and immune checkpoint inhibitors can amplify antitumor efficacy. CXCR4, a GPCR, is overexpressed in cancer cells which mediates cell proliferation, tumor growth, metastasis and tumor relapse [311]. CXCL12, the chemokine binding to the CXCR4, is secreted from fibroblast cells in the tumor microenvironment. CXCL12/CXCR4 axis is another molecular target for cancer treatment as this axis leads to an immunosuppressive tumor microenvironment. Thus, combining plerixafore (AMD3100; CXCR4 inhibitor) with immune checkpoint inhibitor anti-PDL1 antibody showed decreased tumor volume [312].

## Combination immunotherapy with epigenetic drugs

Epigenetic drugs can potentiate combination immunotherapy [313]. DNA hypomethylating agent 5-aza-2′-deoxycytidine (5-AZA-CdR) (a DNA methyltransferase inhibitor, DNMTi) combined with an anti-CTLA-4 monoclonal antibody showed enhanced antitumor efficacy in murine model of breast cancer [314]. In Phase Ib clinical trial, the epigenetic drug guadecitabine was combined with ipilimumab (anti-CTLA-4 antibody) for patients having stage III/IV melanoma. The DNA hypomethylating drugs cause upregulation of genes CD274, PDCD1LG2, and CTLA-4 in the patients where resistance has developed. Currently, a PEG-based nanoparticle system was utilized to deliver plasmid-encoding shPD-L1 (this plasmid down-regulated expresion of PD-L1 protein of cancer cells) in combination with Zebularine (a DNMTi, causing overexpression of major histocompatibility complex I [MHC-I] expression). This combination can effectively initiate anticancer immunity and prevent tumor relapse by a strong anticancer memory [315].

## Limitations and future prospect of nanoparticle-mediated combination immunotherapy

Currently, immunotherapy is the latest approach that has emerged in finding a cure for cancer. However, only immunotherapy is insufficient to eradicate tumors due to : (1) lack of specificity and systemic toxicity, (2) low patient response rate, (3) variable immune contexture of patients, etc., (4) impaired immune function and antibody activity due to the acidic tumor microenvironment (Warburg effect), (5) divergent immune pattern of different organs, (6) development of resistance against immunotherapy, (7) autoimmune adverse effect generation by immune checkpoint blockade, (8) increased immune suppression with aging and (9) huge cost [316–318].

To advance the area of cancer immunotherapy in near future, toxicity issues associated with immunotherapy need to be addressed. Since cancer is a highly heterogeneous disease, nanocarrier encapsulated precision combinatorial immunotherapy will hold a great prospect in future cancer therapy. Tumor heterogeneity can be explored by the determination of biomarkers via genomic analysis of circulating cancer cells. Next-generation sequencing can be deployed to explore immunogenicity of mutated genes. These data along with resources like cBioPortal, Project Genie can be utilized in designing patient-specific immunotherapy in an effective and safer manner. Immunotherapy can be further boosted by utilizing combinatorial approaches. This would require identification of optimal dosing and delivering the same dose to the tumor site. This can be achieved by ratiometric dosing as described earlier. Mono anti-PD-1 therapy often causes compensatory activation of other checkpoints (e.g., CTLA-4), leading to immunosuppression [319]. Thus, simultaneous blocking of inter-related checkpoints is necessary to design effective combinatorial immunotherapy. The gut microbiome or blood supply to the tumor site can also alter responses to cancer immunotherapy by indirectly modifying tumor microenvironment [320]. Hence modulating these factors can help combating resistance to immunotherapy. Furthermore, antagonizing low pH of tumor microenvironment by acidity modulating drugs (proton-pump inhibitors) might act as a possible choice to overcome tumor resistance, potentiating the existing immunotherapy [318]. Thus, optimizing the current immunotherapy with personalized combinatorial nanomedicines can advance the development of next-generation cancer nano-immunotherapy.

## Conclusion and future direction

The challenges of next-generation nanoparticle mediated multicomponent combination therapy and combination immunotherapy need to amplify the therapeutic potential by providing the enhanced stability of cancer drugs in biologically active form, reducing toxicity related issues, overcoming immunosuppression and preferentially accumulating at the tumor site. Due to lack of fenestered or discontinuous endothelium, effective delivery of clinically safe nanomedicine to less accessible tissues remains a considerable challenge. To achieve clinically potential, optimized nanoparticle designing, innovation in designing the mechanistically inspired nanoparticle, targeting specific metastatic foci, their delivery process and *in vivo* biodistribution based on structure and activity need detailed understanding. Integrating cancer biology and anti-metastatic nanotechnology, new strategies need to be engineered considering the biological mechanisms of various stages of metastasis. Tumor microenvironment specific targeted therapeutic intervention is desirable to improve the outcome. New guidelines need to be developed about engineering multicomponent combination therapy and it requires extensive studies on various classes of drug combinations, our understanding about inter-drug interactions for such cases, spatio-temporal release of anticancer drugs, nonspecific activation of the immune system by such multicomponent combination nanomedicine, diversity of metastatic foci, diversity of organ environment and delivery of biologically active multicomponent nanomedicine at the epicentre of solid tumor. Such therapeutics need to be developed by integrating clinical trials with an adequate number of patients having similar biomarker expression and the development of better pre-clinical models. Next-generation multifunctional cancer nanomedicine and nanoparticle mediated combination immunotherapy having mechanistically rational therapeutic combinations need to promote “multi-targeted therapy” by disrupting the adaptive chemoresistance and potentiating the effect of therapeutic combinations. Integrating proteomics data of patient samples, combination nanotherapy and immune-oncology, one may develop highly effective precision nanomedicine.

## Abbreviations

CD47: cluster of differentiation 47
CSF-1R: colony stimulating factor 1 receptor
CTLA-4: cytotoxic T-lymphocyte–associated antigen 4
DNMTi: DNA methyltransferase inhibitor
GPCR: G-protein-coupled receptor
IFN-γ: interferon-γ
MCSF: macrophage colony stimulating factor
MHC-I: major histocompatibility complex I
NIR: near infrared
PD-1: programmed cell death protein 1
PD-L1: programmed death-ligand 1
SIRPα: signal regulatory protein α
TAM: tumor associated macrophages
TGFβ: transforming growth factor-β
